# Control of noise-induced coherent oscillations in three-neuron motifs

**DOI:** 10.1007/s11571-021-09770-2

**Published:** 2021-12-23

**Authors:** Florian Bönsel, Patrick Krauss, Claus Metzner, Marius E. Yamakou

**Affiliations:** 1 Chair for Dynamics, Control and Numerics, Department of Data Science, Friedrich-Alexander-Universität Erlangen-Nürnberg, Cauerstr. 11, 91058 Erlangen, Germany; 2 Biophysics Group, Friedrich-Alexander-Universität Erlangen-Nürnberg, Henkestr. 91, 91052 Erlangen, Germany; 3 Neuroscience Lab, University Hospital Erlangen, Friedrich-Alexander-Universität Erlangen-Nürnberg, Waldstr. 1, 91054 Erlangen, Germany

**Keywords:** Self-induced stochastic resonance, Excitable neurons, Synapses, Autapses, Motif network, Multiplex network

## Abstract

The phenomenon of self-induced stochastic resonance (SISR) requires a nontrivial scaling limit between the deterministic and the stochastic timescales of an excitable system, leading to the emergence of coherent oscillations which are absent without noise. In this paper, we numerically investigate SISR and its control in single neurons and three-neuron motifs made up of the Morris–Lecar model. In single neurons, we compare the effects of electrical and chemical autapses on the degree of coherence of the oscillations due to SISR. In the motifs, we compare the effects of altering the synaptic time-delayed couplings and the topologies on the degree of SISR. Finally, we provide two enhancement strategies for a particularly poor degree of SISR in motifs with chemical synapses: (1) we show that a poor SISR can be significantly enhanced by attaching an electrical or an excitatory chemical autapse on one of the neurons, and (2) we show that by multiplexing the motif with a poor SISR to another motif (with a high SISR in isolation), the degree of SISR in the former motif can be significantly enhanced. We show that the efficiency of these enhancement strategies depends on the topology of the motifs and the nature of synaptic time-delayed couplings mediating the multiplexing connections.

## Introduction

Noise is ubiquitous in biological systems and in particular in neural systems. Contrary to the intuitive perception of noise as deteriorating signal quality (McDonnell and Ward 2011), several studies have shown the constructive effects of noise on neural dynamics (Longtin 1993; Patel and Kosko 2008; Gang et al. 1993; Gutkin et al. 2007), perception and cognition (Krauss et al. 2016, 2017, 2018; Schilling et al. 2020, 2021). The most remarkable of these effects is the phenomenon of noise-induced resonance, in which an optimal amount of noise enhances the detection of weak oscillations and the coherence of these oscillations in a neural systems.

There are several noise-induced resonance phenomena with different emergent conditions and mechanisms, and thus, may play different roles in information processing under different settings of the neural system, see, e.g., Yamakou and Jost (2017), Yamakou and Jost (2019), DeVille et al. (2005) and Zamani et al. (2020). These include, amongst others, the well-known stochastic resonance (SR) (Longtin 1993; Wiesenfeld and Moss 1995; Lindner et al. 2004; Guo et al. 2017; Patel and Kosko 2008; Benzi et al. 1981; Gang et al. 1993), coherence resonance (CR) (Xu et al. 2019; Gang et al. 1993; Pikovsky and Kurths 1997; Lindner and Schimansky-Geier 1999; Gammaitoni et al. 1998; Zhou et al. 2001; Neiman et al. 1997; Zhu 2020), inverse stochastic resonance (ISR) (Gutkin et al. 2007, 2009; Uzuntarla et al. 2013; Yamakou and Jost 2017, 2018), recurrence resonance (RR) (Krauss et al. 2019), and self-induced stochastic resonance (SISR) (Yamakou and Jost 2017; Freidlin 2001a, b; Muratov et al. 2005; DeVille et al. 2005; Muratov and Vanden-Eijnden 2008; DeVille and Vanden-Eijnden 2007a, b; Yamakou and Jost 2018, 2019).

In this work, we focus on SISR. Generically, SISR occurs when a multiple-timescale excitable dynamical system is driven by a noise of weak amplitude. During SISR (see, e.g., Fig. 3b), the escape timescale of trajectories from one attracting region (e.g., the left monotonically decreasing parts of the *S*-shaped nullcline in Fig. 3b) in phase space to another (e.g., the right monotonically decrease parts of the *S*-shape nullcline in Fig. 3b) is exponentially distributed, and the associated transition rate is governed by an activation energy (e.g., the energy barriers $\varDelta U^l_i(w_e)$ and $\varDelta U^r_i(w_{p,i})$ defined in Eq. (11)). If the excitable system (e.g., a neuron) is placed out-of-equilibrium, and its activation energy decreases monotonically as the neuron relaxes slowly to a stable quiescent state (stable fixed point), then at a specific instant during the relaxation, the timescale of escape due to noise and the timescale of relaxation match, and the neuron fires almost surely at this point. If this activation brings the neuron back out-of-equilibrium, the relaxation stage can start over again, and the scenario repeats itself indefinitely, leading to a coherent spiking activity which cannot occur without noise. SISR essentially depends on the interplay of three different timescales: the slow and fast timescales in the deterministic equation of the system, plus a third timescale characteristic to the noise.

In 2005, Muratov et al. (2005) coined the term self-induced stochastic resonance after they discovered the mechanism behind this noise-induced resonance phenomena in a chemical model equation. After the 2005 paper, a series of papers on SISR in other models including neural systems (DeVille et al. 2005; Muratov and Vanden-Eijnden 2008; Yamakou and Jost 2018, 2019; Yamakou et al. 2020), Brownian ratchets (DeVille and Vanden-Eijnden 2007b), cancer model (Shen et al. 2010), and even in bearing faults model (Zhang et al. 2021a) were published, each showing how generic the mechanism of SISR in a slow-fast stochastic excitable system is, and how ubiquitous it is in physical, biological, and chemical systems.

All previous studies have investigated SISR in isolated oscillators, except in the case of neural systems, where only two studies have investigated SISR in networks of coupled neurons (Yamakou and Jost 2019; Yamakou et al. 2020). It was shown in Yamakou and Jost (2019) that, in contrast to SISR in a single isolated FitzHugh-Nagumo (FHN) neuron, the maximum noise amplitude at which SISR can occur in the network of coupled FHN neurons is not fixed (i.e., is controllable), especially in the regime of strong synaptic couplings and long time delays. And in Yamakou et al. (2020), the performance of electrical and inhibitory chemical synapses in the enhancement of the degree of SISR in layer and multiplex networks of FHN neurons are compared. It was shown that for each isolated layer network, weaker electrical and chemical synaptic couplings are better enhancers of SISR. It was also shown that, regardless of the synaptic strengths, shorter electrical synaptic delays are better enhancers of SISR than shorter chemical synaptic delays, while longer chemical synaptic delays are better enhancers than longer electrical synaptic delays. Furthermore, it is found that electrical, inhibitory, or excitatory chemical multiplexing of the two layers having only electrical synapses at the intra-layer levels can each enhance SISR. Additionally, only excitatory chemical multiplexing of the two layers having only inhibitory chemical synapses at the intra-layer levels can enhance SISR. Furthermore, in Yamakou and Jost (2019) and Yamakou et al. (2020), the enhancement of SISR is based on the configuration of the electrical and chemical synapses between the connected neurons within a layer network and between layers in a multiplex network. No studies have reported on the (in)efficiency of autapses—self-feedback synapses—on the enhancement of SISR in neurons. The current work aims at bridging this gap.

Moreover, in all previous studies of SISR in neural systems, including isolated neurons (Yamakou and Jost 2017; DeVille et al. 2005; Muratov and Vanden-Eijnden 2008; Yamakou and Jost 2018; Yamakou and Tran 2020; Zhu and Nakao 2021) and neural networks (Yamakou and Jost 2019; Yamakou et al. 2020), the mathematically simpler but biophysically less realistic FHN neuron model has been used. In this work and for the very first time, we study SISR and its control in a conductance-based neuron model, i.e., in the more realistic Morris–Lecar (ML) model (Morris and Lecar 1981). The mathematical structure of the ML neuron model—low dimensional, existence of a (explicit) strong timescale separation between the dynamical variables (conditions required SISR), and to some degree, a tractable nonlinear vector field—makes it a perfect conductance-based model for the analysis of SISR.

In information processing, networks take different tasks of functionality (Markram 2012; Van Essen et al. 2013). Thus, a better understanding of their structure and connectivity should shed more light on the dynamics of the phenomena occurring on them (Krauss et al. 2019). It is well-known that large recurrent networks can be decomposed into smaller building blocks—the so-called motifs (Milo et al. 2002), whereby three-neuron motifs are the most basic motifs, which frequently appear in neural circuits and can be seen as basic computational units (Krauss et al. 2019), each uniquely contributing to a large-scale neural behavior (Li 2008; Song et al. 2005). Thus, in this paper we focus on these basic computational units—three-neuron motifs (3NMs). Another important class of networks is the so-called multiplex network. It consists of two or several layer networks connected to each other, with each node in one layer connected only to its replica node in another layer (Battiston 2017). This kind of inter-layer coupling can induce complex behaviors, namely: the emergence and suppression of chimera states (Maksimenko et al. 2016; Ghosh et al. 2016; Ghosh and Jalan 2016), and the formation of synchronization patterns (Sawicki et al. 2018; Goremyko et al. 2017), including intra-layer synchronization effects (Goremyko et al. 2017; Singh et al. 2015). It has been shown that multiplexing of layer networks can be used to control the dynamics of one layer by tuning the parameters of another layer. For example, multiplexing of layer networks has been shown to be an efficient strategy for improving CR in one layer of a two-layer multiplex network by tuning the parameters of the other layer Semenova and Zakharova (2018); Yamakou and Jost (2019) network. In particular, it was found that multiplexing can induce CR in a layer which does not exhibit this phenomenon in isolation. Moreover, it has been shown that the control of CR can be achieved even for weak multiplexing. While these theoretical results are intriguing, it remains an open question to which extent they affect our understanding of the neural information processing underlying perception, cognition, and behavior in biological organisms. In the case of multiplexing, for example, a fundamental question is where the required point-to-point connections might be found in actual nervous systems. Possible candidates are the nerve fibres connecting the layers within cortical micro columns (Kandel et al. 2000), or also the commissural fibers of the corpus callosum, which are known to form point-to-point connections between homologous cortex areas in the two different hemispheres (Aboitiz et al. 1992; Schüz and Preibl 1996). Even if these anatomical structures can be interpreted as cases of multiplexing, it must however be assumed that heterogeneous multiplex congurations, i.e., those between dierent types of motifs, are signicantly more likely than homogeneous congurations of identical motifs. We therefore consider in this work also the SISR phenomenon in systems of two structurally different, but mutually coupled motifs.

Due to the complexity of noise-induced resonance phenomena, most existing studies are forced to consider relatively small networks of idealized model neurons, and they are typically based on assumptions that make an extrapolation of the obtained results to larger neural networks quite difficult. For example, while many theoretical works consider electrical synapses, chemical synapses are by far the most common connections between neurons in the brain (Pereda 2014), and particularly in the mammalian central nervous system, their number exceeds that of electrical synapses by several orders of magnitude (Kandel et al. 2000). For this reason, we are considering both electrical and chemical synapses in this work. Moreover, we investigate the effect of so-called autapses (Van Der Loos and Glaser 1972). Autapses are synaptic contacts of a neuron’s axon onto its own dendrite and soma. In the neocortex, self-inhibiting autapses in GABAergic interneurons are abundant in number and play critical roles in regulating spike precision and network activity (Lübke et al. 1996; Yilmaz et al. 2016; Herrmann and Klaus 2004; Guo et al. 2016; Yin et al. 2018; Bacci and Huguenard 2006). Anatomical observations suggest that autapses might be used as compensatory replacements for injured axons (Wang et al. 2017), or to enhance persistent neural activity (that is supposed to be) elementary for short-term memory storage (Seung et al. 2000). Some research papers have shown that autapses can significantly influence the dynamics of single-neurons (Wang et al. 2014; Liu and Yang 2018) and neural networks, including synchronization (Protachevicz et al. 2020; Fan et al. 2018), SR (Yang et al. 2017), CR (Yilmaz et al. 2016; Song et al. 2018; Jia et al. 2021), and ISR (Zhang et al. 2021). However, as we pointed out earlier, no study have investigated the effects of autapses on SISR and how these effects can be combined with the network topology to enhance the coherence of oscillations induced by SISR—this is one objective of the current paper.

Furthermore, Fries (2005) suggested that coherence of neural activity is conducive to neural communication. For instance, it was demonstrated that coherence is advantageous for the signal transmission between spatially separated active brain regions (Benchenane et al. 2010). This communication can be achieved simultaneously at different ranges of oscillation frequency (Fries 2015), which would not be possible if they show incoherent behavior. Motivated by these studies, in this work, we focus on SISR in single ML neurons, 3NMs of time-delayed coupled neurons, and how it can be controlled through autapses (Van Der Loos and Glaser 1972; Lübke et al. 1996) and multiplexing (Battiston 2017). In this paper, we address the following four main questions:

1. How does the type of autaptic connections affect the degree of SISR in a single-ML neuron?
2. How does the type of synaptic connections and topology of a motif affect SISR?
3. Can a poor degree of SISR in a motif be enhanced by autapses?
4. Can a poor degree of SISR in a motif be enhanced by multiplexing?

The rest of the paper is organized as follows: In Sect. 2, we introduce the model equations. In Sect. 3, we represent the analytical conditions necessary for the occurrence of SISR in the model. In Sect. 4, we represent the numerical methods used in our simulations. In Sect. 5, we present and discuss the simulation results and in Sect. 6, we have a summary with concluding remarks.

## Model description

Different neural models have been used to investigate several dynamical behaviors ranging from synchronization (Wouapi et al. 2020, 2021; Boaretto et al. 2021; Yu et al. 2021) to resonance (Masoliver et al. 2017; Liu and Yang 2018; Lu et al. 2020; Wang et al. 2021). In this paper, we consider a network of ML neurons with type-II excitability and driven by Gaussian processes to investigate SISR. The network is described by the following coupled stochastic delayed differential equations:

$$\begin{aligned} \begin{aligned} \left\{ \begin{array}{lcll} \frac{dv_{p,i}}{dt} =& f(v_{p,i},w_{p,i}) + \sigma _{p,i} \frac{dW_{p,i}}{dt} &{} ({\mathrm{1a}}) \\ &\quad +\kappa ^a_{\mathrm {e}}f^a_e(v_{p,i})+\kappa ^a_{c}f^a_{c}(v_{p,i})&\quad ({\mathrm{1b}})\\ &\quad + \kappa _{\mathrm {e}}f^G_e(v_{p,i},v_{p,j})+\kappa _{c}f^G_{c}(v_{p,i},v_{p,j})&{} ({\mathrm{1c}})\\ &\quad + \kappa ^m_{\mathrm {e}}f^m_e(v_{p,i}, v_{q,i}) +\kappa ^m_{c}f^m_{c}(v_{p,i}, v_{q,i}), &{} ({\mathrm{1d}}) \\ \frac{dw_{p,i}}{dt} =& \varepsilon g(v_{p,i},w_{p,i}). &{} ({\mathrm{1e}}) \end{array}\right. \end{aligned} \end{aligned}$$

Here, the membrane potential and the recovery current variables of neuron *i* in the motif layer *p* are given by $v_{p,i}\in \mathbb {R}$ and $w_{p,i}\in \mathbb {R}$, respectively. To avoid confusion, one may keep in mind that the first indices, i.e., $p,q\in \{1,2\}$ ($p\ne q$), denote the motif layer in which the neuron is located, while the second indices, i.e., $i,j \in \{1,2,3\}$ ($i\ne j$), denote the *i*th and *j*th neuron within a given layer: *p* or *q*.

Eq. (1) in the absence of Eqs. (1b), (1c), and (1d), represents a single isolated stochastic ML neuron without autapses, where the deterministic nonlinear vector fields $f(v_{p,i},w_{p,i})$ in Eq. (1a) and $g(v_{p,i},w_{p,i})$ in Eq. (1e) are, after dropping the indices, given by

2 $$\begin{aligned} \begin{aligned} \left\{ \begin{array}{lcl} f(v,w)&{}=&{} \overline{g}_c m_{\infty }(v)(1-v) + \overline{g}_l(v_l-v)\\ &{}+&{}\overline{g}_k w (v_k-v),\\ g(v,w) &{}=&{} \displaystyle {\mathrm {cosh}\left( \frac{v-v_3}{v_4} \right) \left( w_{\infty }(v)- w\right) }, \end{array}\right. \end{aligned} \end{aligned}$$

where the nonlinearities get in via $m_{\infty }(v)$ and $w_{\infty }(v)$, each given by

3 $$\begin{aligned} \begin{aligned} \left\{ \begin{array}{lcl} m_{\infty }(v)= \displaystyle {\frac{1}{2}\left[ 1+ \mathrm {tanh}\left( \frac{v-v_1}{v_2} \right) \right] },\\ w_{\infty }(v)= \displaystyle {\frac{1}{2}\left[ 1+ \mathrm {tanh}\left( \frac{v-v_3}{v_4} \right) \right] }, \end{array}\right. \end{aligned} \end{aligned}$$

with $\overline{g}_c=1.0$, $\overline{g}_k=1.0$, and $\overline{g}_l=0.1$ representing the conductances; and $v_k=-2.0$, $v_1=0.0$, $v_2=0.36$, $v_3=-0.2$, and $v_4=0.52$ representing constant parameters (Liu et al. 2014). The excitability parameter $v_l$ is a codimension-one Hopf bifurcation parameter for the ML neuron. And $0<\varepsilon \ll 1$ is a small positive parameter that sets the timescale separation between the fast membrane potential and the slow recovery current variables.

$dW_{p,i}/dt$ are uncorrelated Gaussian white noises, that is the formal derivative of Brownian motion with $\langle dW_{p,i}(t),dW_{p,i}(t')\rangle _t =\delta (t-t')$ and variance (intensity) $\sigma _{p,i}$. For the sake of simplicity, we assume that the noise intensities $\sigma _{p,i}$ within a given layer *p* are all the same, i.e., we choose $\sigma _{p,1}=\sigma _{p,2}=\sigma _{p,3}$. The parameters $\sigma _{p,i}$ and $\varepsilon$ are crucial for the occurrence of SISR.

**Fig. 1:**
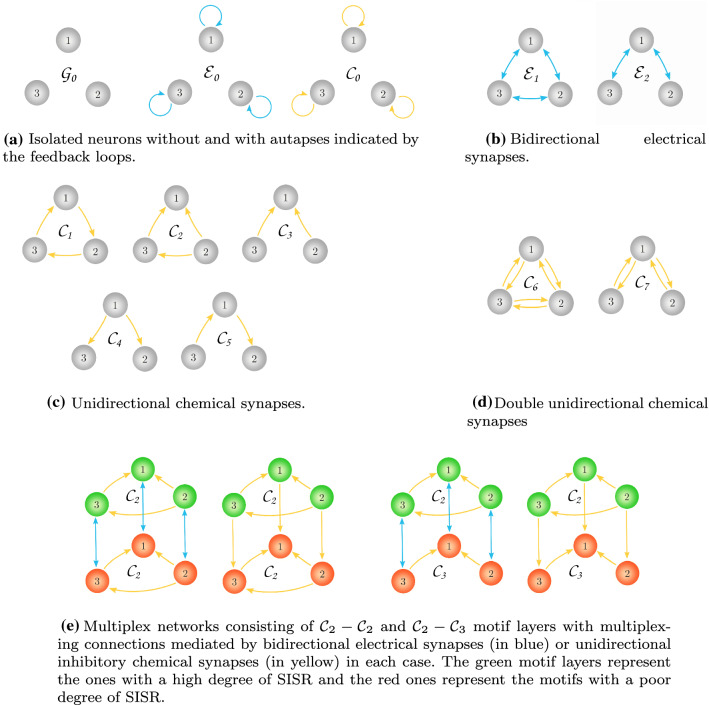
Schematic representations of the network topologies investigated in this work, indicating the types and configurations of the synaptic connections

Figure 1 shows the specific motif layer networks and the multiplex network configurations, including isolated neurons, that will be considered in this work. In Eq. (1b), $\kappa ^a_{\mathrm {e}}f^a_e(v_{p,i})$ and $\kappa ^a_{c}f^a_{c}(v_{p,i})$ respectively represent the electrical and chemical autaptic terms of the *i*th neuron in the *p*th layer. Here, $\kappa ^a_{\mathrm {e}}$ represents the strength of the electrical autapse and $\kappa ^a_{c}$ the strength of the chemical autapse. Furthermore, $f^a_e(v_{p,i})$ and $f^a_c(v_{p,i})$ are respectively given by the well-known (Iqbal et al. 2017; Wang et al. 2006; Xu et al. 2017; Destexhe et al. 1994, 1998; Greengard 2001) forms of the electrical and chemical autapses:

4 $$\begin{aligned} \begin{aligned} \left\{ \begin{array}{lcl} f^a_e(v_{p,i}) &{}=&{} \big (v_{p,i}(t-\tau ^a_e) - v_{p,i}(t)\big ),\\ f^a_c(v_{p,i}) &{}=&{} \displaystyle { \frac{\big (v_{p,i}(t)-v_{\mathrm {syn}}\big )}{1+ e^{-\lambda \big [v_{p,i}(t-\tau ^a_c) -\theta _{\mathrm {syn}}\big ]}}}. \end{array}\right. \end{aligned} \end{aligned}$$

In Eq. (1c), $\kappa _{\mathrm {e}}f^G_e(v_{p,i},v_{p,j})$ and $\kappa _{c}f^G_{c}(v_{p,i},v_{p,j})$ respectively represent the electrical interaction (gap junctions) between the *i*th and *j*th neurons in layer *p* and the chemical interaction from the *i*th to the *j*th neurons in the *p*th layer. Here, $\kappa _{\mathrm {e}}$ and $\kappa _{c}$ represents the strength of the electrical and chemical couplings, respectively. The terms $f^G_{\mathrm {e}}(v_{p,i},v_{p,j})$ and $f^G_{c}(v_{p,i},v_{p,j})$ are given by

5 $$\begin{aligned} \begin{aligned} \left\{ \begin{array}{lcl} f^G_e(v_{p,i},v_{p,j}) &{}=&{}\sum \limits _{j\ne i}\mathcal {G}_{ij}\big (v_{p,i}(t-\tau _e) - v_{p,j}(t)\big ),\\ f^G_c(v_{p,i},v_{p,j}) &{}=&{}\sum \limits _{j\ne i}\mathcal {G}_{ij}\displaystyle { \frac{\big (v_{p,i}(t)-v_{\mathrm {syn}}\big )}{1+e^{-\lambda \big [v_{p,j}(t-\tau _c) -\theta _{\mathrm {syn}}\big ]}}}. \end{array}\right. \end{aligned} \end{aligned}$$

In Eq. (1d), $\kappa ^m_{\mathrm {e}}f^m_e(v_{p,i}, v_{q,i})$ and $\kappa ^m_{c}f^m_{c}(v_{p,i},v_{q,i})$ respectively represent the electrical and chemical interactions (multiplexing) between the *i*th neuron in layer *p* and the *i*th neuron in layer *q*. That is, in multiplex networks, connections exist only between replica neurons. Similarly, $\kappa ^m_{\mathrm {e}}$ and $\kappa ^m_{c}$ represent the strengths of the electrical and chemical multiplexing, respectively. Here, $f^m_e(v_{p,i}, v_{q,i})$ and $f^m_{c}(v_{p,i},v_{q,i})$ are given by

6 $$\begin{aligned} \begin{aligned} \left\{ \begin{array}{lcl} f^m_e(v_{p,i},v_{q,i}) &{}=&{}\big (v_{q,i}(t-\tau ^m_e) - v_{p,i}(t)\big ),\\ f^m_c(v_{p,i},v_{q,i}) &{}=&{}\displaystyle { \frac{\big (v_{p,i}(t)-v_{\mathrm {syn}}\big )}{1+e^{-\lambda \big [v_{q,i}(t-\tau ^m_c) -\theta _{\mathrm {syn}}\big ]}}}. \end{array}\right. \end{aligned} \end{aligned}$$

It is worth noting from Fig. 1 and Eqs. (5) and (6) that the electrical synapses are always bidirectional (represented in Fig. 1b by the blue links with double arrow going from the *i*th to the *j*th neuron and also back, i.e., from the *j*th to the *i*th neuron). On the other hand, chemical synapses can either be (1) a single unidirectional connection (i.e., for Eq. (5), it is represented in Fig. 1c by the yellow links with a single arrow from the *i*th to the *j*th neuron; and for Eq. (6), it is represented in Fig. 1e by the single yellow links with a single arrow going from the *i*th neuron in the *p*th layer to the *i*th neuron in the *q*th layer) or (2) a double unidirectional connection (i.e., for Eq. (5), it is represented in Fig. 1d by two yellow links each representing a single unidirectional chemical connection, one going from the *i*th to the *j*th neuron and the other from the *j*th to the *i*th neuron. This kind of reciprocal connection is a very common and universal design principle of biological nervous systems (Markram 1997; Pitkänen et al. 2000; Song et al. 2005; Zupanc and Corrêa 2005; Perin et al. 2011; Bastos et al. 2012). For Eq. (6) (i.e., the coupling terms between two motif layers) we consider one chemical unidirectional connection from the *i*th neuron in the *p*th layer to the *i*th neuron in the *q*th layer and the other from the *i*th neuron in the *q*th layer to the *i*th neuron in the *p*th layer). The electrical and chemical autapses given by Eq. (4) are self-loops on each neuron and are represented in Fig. 1a by the blue and yellow loops, respectively.

In Eq. (4), $\tau ^a_e$ and $\tau ^a_c$ respectively represent the electrical and chemical autaptic time delays. In Eq. (5), $\tau _e$ and $\tau _c$ respectively represent the time delays involved in the electrical and chemical interactions between neurons within the same layer. While in Eq. (6), $\tau ^m_e$ and $\tau ^m_c$ represent the electrical and chemical multiplexing time delays, respectively. Moreover, the parameter $\lambda$ (which is fixed at $\lambda =5.0$ in this work) determines the slope of the sigmoidal input-output function $\Gamma (v)=\displaystyle { 1/(1+e^{-\lambda (v -\theta _{\mathrm {syn}})}})$, where $\theta _{\mathrm {syn}}$ represents the synaptic firing threshold (which is fixed at $\theta _{\mathrm {syn}}=0.0$ in this work). And $v_{\mathrm {syn}}$ represents the synaptic reversal potential.

When $v_{\mathrm {syn}} < v_{p,i}$, the chemical interaction has a depolarizing effect which makes the synapse inhibitory, and when $v_{\mathrm {syn}} > v_{p,i}$, the chemical interaction has a hyper-polarizing effect, making the synapse excitatory. This means that we can choose a value for $v_{\mathrm {syn}}$ such that the inhibitory and excitatory nature of the chemical synapse is determined only by the sign in front of the chemical coupling strengths $\kappa ^a_c$, $\kappa _c$, and $\kappa ^m_c$. For the ML neuron model used in this study, the membrane potential variables are certainly bounded as: $-1.4<v_{p,i}(t)<2.5$ ($p = 1,2$; $i = 1, 2, 3$) for all time t. We fix $v_{\mathrm {syn}} = -1.5$ (maintained throughout our computations), a value with which the term $(v_{p,i}(t) - v_{\mathrm {syn}})$ is always positive. In this way, a positive sign in front of $\kappa ^a_c$, $\kappa _c$ and $\kappa ^m_c$ will always make these chemical synapses excitatory, represented by $\kappa ^a_{\mathrm {c,exc}}$, $\kappa _{\mathrm {c,exc}}$, and $\kappa ^m_{\mathrm {c,exc}}$, respectively. While a negative sign in front of $\kappa ^a_c$, $\kappa _c$ and $\kappa ^m_c$ will always make these chemical synapses inhibitory, represented $\kappa ^a_{\mathrm {c,inh}}$, $\kappa _{\mathrm {c,inh}}$, and $\kappa ^m_{\mathrm {c,inh}}$, respectively.

The matrix $\mathcal {G}$ in Eq. (5) represents the adjacency matrix of a motif layer network. The entry $\mathcal {G}_{ij}$ is 1 if the *i*th neuron is connected to the *j*th neuron and 0 otherwise.

## Deterministic predisposition and necessary conditions for SISR

A ML neuron with a unique and stable fixed point and in the complete absence of random perturbations (or even in the presence of a sub-threshold deterministic perturbation) cannot maintain a self-sustained oscillation (i.e., no limit cycle solution can emerge). One says in this case that the neuron is in the excitable regime (Izhikevich 2000). The predisposition state for the occurrence of SISR in an isolated neuron and in a network of neurons is precisely excitability. In an excitable state, choosing an initial condition in the basin of attraction of the unique and stable fixed point will result in at most one large non-monotonic excursion in the phase space after which the trajectory returns exponentially fast to this fixed point and stays there until a disturbance like a random perturbation is introduced in the neuron model.

**Fig. 2:**
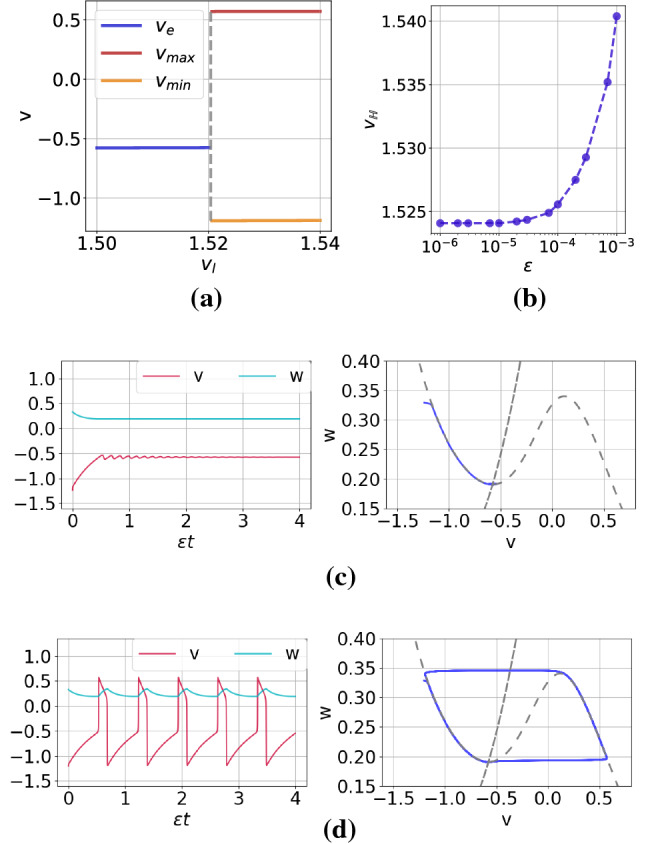
Panel **a**: Bifurcation diagram of a single isolated ML neuron without autapses against the excitability parameter $v_l$ at a fixed timescale separation parameter $\varepsilon =0.0005$. The Hopf bifurcation value separating the excitable regime (blue line) and oscillatory regime (orange line) is indicated by the dashed-gray vertical line located at $v_l=v_{\mathbb {H}}(\varepsilon =0.0005)=1.52010$. Panel **b**: Variation of the Hopf bifurcation value with the timescale parameter $\varepsilon$. To avoid oscillatory regime due to Hopf bifurcation, we fixed $v_l=1.515$ in all simulations. Panels **c** and **d** show, each, a time series (left) and the associated phase portrait (right) of a trajectory in the excitable regime $v_l=1.515<v_{\mathbb {H}}$ and in the oscillatory regime $v_l=1.525>v_{\mathbb {H}}$, respectively. (Color figure online)

In a single isolated ML neuron without autapses, the excitability parameter is $v_l$. Figure 2a shows a bifurcation diagram of a single ML neuron without autapses at a fixed timescale separation parameter $\varepsilon =0.0005$. It is worth noting that the real part of the eigenvalue of the Jacobian matrix associated to a single isolated ML without autapses depends on the timescale separation parameter $\varepsilon$. Hence, the Hopf bifurcation value of the isolated ML neuron without autapses also depends on $\varepsilon$. We observe that by varying the Hopf bifurcation parameter $v_l$ in the interval [1.50, 1.52010), the membrane potential *v* stays at a constant value (i.e., at the unique and stable fixed point $v_e$) represented by the blue horizontal line. At the Hopf bifurcation value $v_l=v_{\mathbb {H}}(\varepsilon =0.0005)=1.52010$, the stable and unique fixed point bifurcates into a stable limit cycle, represented by the dashed-gray vertical line. The top and bottom orange horizontal lines represent the maximum and the minimum values of the limit cycle oscillation, respectively.

Figure 2b shows the variation of the Hopf bifurcation value $v_{\mathbb {H}}$ with timescale separation parameter $\varepsilon$. We observe that the Hopf bifurcation value is non-linearly proportional to $\varepsilon \ll 1$, and for $10^{-6}\le \varepsilon \le 10^{-5}$, the Hopf bifurcation value remains constant at $v_{\mathbb {H}}=1.524$. For this reason, we keep the isolated ML neuron without autapses in the excitable regime by fixing $v_l$ at $v_l=1.515$ throughout this work, so that we are sure that coherent oscillations due to a Hopf bifurcation cannot occur in our simulations. Figure 2c and d show the time series and the corresponding phase portrait of a trajectory when we choose $v_l$ in the excitable regime and in the oscillatory regime, respectively. In the phase portraits, the *S*-shaped curve corresponds to the *v*-nullcline which intersects the *w*-nullcine at a single point, i.e., the unique and stable (unstable in Fig. 2d) fixed point.

It is worth pointing out that in the deterministic single isolated ML neuron with autapse and the deterministic networks of ML neurons with and without autapses, pairs of new parameters, i.e., $(\kappa ^a_e, \tau ^a_e)$, $(\kappa ^a_c, \tau ^a_c)$, $(\kappa _e, \tau _e$), $(\kappa _c, \tau _c)$, $(\kappa ^m_e, \tau ^m_e)$, $(\kappa ^m_c, \tau ^m_c)$, enter the bifurcation dynamics. It is therefore very important to also check that, after fixing $v_l=1.515$, these additional set of parameters does not shift the deterministic system into the oscillatory regime via Hopf or saddle-node onto limit cycle bifurcations (Schöll et al. 2009; Yamakou and Jost 2019; Yamakou et al. 2020).

We recall that SISR is the occurrence of a limit cycle behavior (coherent oscillations) due to solely the presence of noise and not because of the occurrence of deterministic bifurcations onto limit cycles. To avoid such deterministic oscillatory regimes, we will always first check that the values of all autaptic, intra-motif, and inter-motif delayed coupling parameters keep the considered system in the excitable regime.

The adiabatic limit $\varepsilon \rightarrow 0$ in Eq. (1) reduces Eq. (1e) to $dw_{p,i}/dt\approx 0$. This means that in this limit, the $w_{p,i}$-variables of neurons are frozen, reducing Eq. (1) into a set of coupled Langevin equations given by

7 $$\begin{aligned} \frac{dv_{p,i}}{dt} = -\frac{\partial U(v_{p,i},w_{p,i})}{\partial v_{p,i}} + \sigma _{p,i} \frac{dW_{p,i}}{dt}, \end{aligned}$$

where $U(v_{p,i},w_{p,i})$ is the double well potential defined in Eq. (9), and in which $w_{p,i}$ is essentially constant.

The conditions (based on large deviation theory Freidlin 2001b, a and Kramers’ law 1940) necessary for the occurrence of SISR in stochastic slow-fast dynamical systems in the form of Eq. (1) (the so-called standard form Kuehn 2015) are well established (Muratov et al. 2005; DeVille and Vanden-Eijnden 2007a; Lee DeVille et al. 2005; Yamakou and Jost 2019; Yamakou et al. 2020) and are quite generic for Gaussian noise.

In Eq. (8), we write down these conditions (see, e.g., Yamakou and Jost 2019) for the motif layer *p* of Eq. (1):

8 $$\begin{aligned} \begin{aligned} \left\{ \begin{array}{lcl} v_l-v_{\mathbb {H}}<0,\\ \displaystyle {\lim \limits _{(\sigma _{p,i},\varepsilon )\rightarrow (0,0)}\bigg (\frac{\sigma _{p,i}^2}{2}\ln (\varepsilon ^{-1})\bigg )\in \Big [\varDelta U^l_i(w_e), F_p(\cdot )\Big ]},\\ \displaystyle {\lim \limits _{(\sigma _{p,i},\varepsilon )\rightarrow (0,0)}\bigg (\frac{\sigma _{p,i}^2}{2}\ln (\varepsilon ^{-1})\bigg )=\mathcal {O}(1)}, \end{array}\right. \end{aligned} \end{aligned}$$

where $F_p$, defined in Eq. (10), is a function of the parameters $(\kappa ^a_e, \tau ^a_e)$, $(\kappa ^a_c, \tau ^a_c)$, $(\kappa _e, \tau _e)$, $(\kappa _c, \tau _c)$, $(\kappa ^m_e, \tau ^m_e)$, $(\kappa ^m_c, \tau ^m_c)$; and $\varDelta U^l_i(w_e)$, defined in Eq. (11), is the left energy barrier (as opposed to the right energy barrier $U^r_i(w_{p,i})$, both obtained in the adiabatic limit $\varepsilon \rightarrow 0$) of a double well potential $U(v_{p,i},w_{p,i})$ (see Eq. (9)) at the $w_{p,i}$-coordinate of the unique and stable fixed point $(v_e, w_e)$ of Eq. (1), in the absence of noise.

9 $$\begin{aligned} \begin{aligned} U(v_{p,i},w_{p,i})=&-\int \Big [ f(v_{p,i},w_{p,i}) +\kappa ^a_{\mathrm {e}}f^a_e(v_{p,i})\\&\quad+\kappa ^a_{c}f^a_{c}(v_{p,i}) + \kappa _{\mathrm {e}}f^G_e(v_{p,i},v_{p,j}) \\&\quad+ \kappa _{c}f^G_{c}(v_{p,i},v_{p,j}) + \kappa ^m_{\mathrm {e}}f^m_e(v_{p,i}, v_{q,i})\\ &\quad+ \kappa ^m_{c}f^m_c(v_{p,i}, v_{q,i}) \Big ]dv_{p,i}. \end{aligned} \end{aligned}$$

10 $$\begin{aligned} \begin{aligned} F_p&:=\Big \{(\kappa ^a_e, \tau ^a_e), (\kappa ^a_c, \tau ^a_c), (\kappa _e, \tau _e),(\kappa _c, \tau _c),\\ {}&(\kappa ^m_e, \tau ^m_e), (\kappa ^m_c, \tau ^m_c) : \varDelta U^l_i(w_{p,i}) = \varDelta U^r_i(w_{p,i})\Big \}, \end{aligned} \end{aligned}$$

where

11 $$\begin{aligned} \begin{aligned} \left\{ \begin{array}{lcl} \varDelta U^l_i(w_{p,i})&{}:=&{} U\big (v^{*}_{0}(w_{p,i}),w_{p,i}\big ) - U\big (v^{*}_{l}(w_{p,i}),w_{p,i}\big ),\\ \varDelta U^r_i(w_{p,i})&{}:=&{} U\big (v^{*}_{0}(w_{p,i}),w_{p,i}\big ) - U\big (v^{*}_{r}(w_{p,i}),w_{p,i}\big ), \end{array}\right. \end{aligned} \end{aligned}$$

with

12 $$\begin{aligned} \begin{aligned} v^{*}_{l,0,r}(w_{p,i}):=&\Big \{v_{p,i}: f(v_{p,i},w_{p,i}) +\kappa ^a_{\mathrm {e}}f^a_e(v_{p,i})\\&\quad+\kappa ^a_{c}f^a_{c}(v_{p,i}) + \kappa _{\mathrm {e}}f^G_e(v_{p,i},v_{p,j})\\&\quad+\kappa _{c}f^G_{c}(v_{p,i},v_{p,j}) + \kappa ^m_{\mathrm {e}}f^m_e(v_{p,i}, v_{q,i})\\&\quad+\kappa ^m_{c}f^m_{c}(v_{p,i}, v_{q,i})=0 \Big \}. \end{aligned} \end{aligned}$$

The sets of solution $v^{*}_{l}(w_{p,i})$, $v^{*}_{0}(w_{p,i})$, and $v^{*}_{r}(w_{p,i})$ in Eq. (12) are such that $v^{*}_{l}(w_{p,i})<v^{*}_{0}(w_{p,i})<v^{*}_{r}(w_{p,i})$, define the left stable, middle unstable, and right stable branches of the *S*-shaped *v*-nullcline of the ML neuron model, respectively.

The theoretical result given in Eq. (8) can briefly be interpreted as follow: the first expression (i.e., $v_l\,-\,v_{\mathbb {H}}\,<\,0$) requires the system to be in the excitable regime, i.e., a parameter regime where the zero-noise (deterministic) dynamics does not display a limit cycle nor even its precursor. This condition means that SISR can arise when the parameters are bounded away from bifurcation thresholds (this is in contrast to CR, see, e.g., DeVille et al. (2005); Yamakou and Jost (2019)). The second expression in Eq. (8) shows that the coherence (regularity) of the spiking created by the noise has a non-trivial dependence on the noise amplitude and the time-scale ratio between fast excitatory variables and slow recovery variables. This expression means that the spiking of the neural system will become more coherent if in the double limit $(\sigma _{p,i},\varepsilon ) \rightarrow (0,0)$, the quantity $\big [\frac{\sigma _{p,i}^2}{2}\ln (\varepsilon ^{-1})\big ]$ stays within the interval $\big [\varDelta U^l_i(w_e), F_p(\cdot )\big ]$. The last expression of Eq. (8) requires that, in the double limit $(\sigma _{p,i},\varepsilon ) \rightarrow (0,0)$, the quantity $\big [\frac{\sigma _{p,i}^2}{2}\ln (\varepsilon ^{-1})\big ]$ be as far away as possible from the boundaries of this interval. This last requirement ensures that the trajectories do not spend too much time in the wells of the double-well potential given in Eq. (9), and hence destroy the regularity of the spiking. Therefore, the non-occurrence or strength of SISR, if it occurs, depends on whether (or to what extend) the chosen values of the system parameters $\{(\kappa ^a_e,\tau ^a_e), (\kappa ^a_c,\tau ^a_c), (\kappa _e, \tau _e), (\kappa _c, \tau _c), (\kappa ^m_e, \tau ^m_e), (\kappa ^m_c, \tau ^m_c) \}$ satisfies the expressions in Eq. (8) in the double limit $(\sigma _{p,i},\varepsilon ) \rightarrow (0,0)$.

Using the theoretical result in Eq. (8), we calculate the minimum ($\sigma _{min}$) and maximum ($\sigma _{max}$) noise amplitude between which the degree of SISR is high as follows:

13 $$\begin{aligned} \begin{aligned} \left\{ \begin{array}{lcl} \sigma _{min}=\displaystyle {\sqrt{\frac{2\varDelta U^l_i(w_e)}{\ln (\varepsilon ^{-1})}}},\\ \sigma _{max}=\displaystyle {\sqrt{\frac{2F(\cdot )}{\ln (\varepsilon ^{-1})}}}, \end{array}\right. \end{aligned} \end{aligned}$$

where $\sigma _{min}$ and $\sigma _{max}$ get their dependence on the parameters $(\kappa ^a_e, \tau ^a_e)$, $(\kappa ^a_c, \tau ^a_c)$, $(\kappa _e, \tau _e)$, $(\kappa _c, \tau _c)$, $(\kappa ^m_e, \tau ^m_e)$, and $(\kappa ^m_c, \tau ^m_c)$ from $U(v_{p,i},w_{p,i})$ and $v^{*}_{l,0,r}(w_{p,i})$. Later, we shall return to Eq. (13), when we will use the expressions of $\sigma _{min}$ and $\sigma _{max}$ to provide theoretical explanations to some of our numerical results.

It is also worth noting that the corresponding conditions for the occurrence of SISR in a single isolated neuron with or without autapses and in a single isolated motif layer network without multiplexing can be easily obtained by setting the corresponding autaptic ($\kappa ^a_e$ and/or $\kappa ^a_c$) and multiplexing ($\kappa ^m_e$ and/or $\kappa ^m_c$) coupling strengths in Eqs. (8)–(12) to zero.

To answer the main questions we are interested in (see the introductory section), we fix $v_l=1.515<v_{\mathbb {H}}$ and choose the relevant coupling strengths and the associated time delays such that Eq. (1) is in the excitable regime. We also choose a sufficiently small timescale separation parameter, i.e., $\varepsilon =0.0005\ll 1$, a weak noise intensity interval, i.e., $0<\sigma _{p,i}\ll 1$, and then numerically identify the combined values of $\{(\kappa ^a_e, \tau ^a_e), (\kappa ^a_c, \tau ^a_c), (\kappa _e, \tau _e), (\kappa _c, \tau _c), (\kappa ^m_e, \tau ^m_e), (\kappa ^m_c, \tau ^m_c)\}$ which satisfy (or at least to some degree) or not the scaling limit conditions in Eq. (8).

## Numerical method for integration

In this work, the coefficient of variation ($\mathrm {CV}$) (Pikovsky and Kurths 1997; Masoliver et al. 2017) will be used to measure the degree of coherence of spiking induced via the mechanism of SISR and hence, the extent of satisfaction of Eq. (8), when the various synaptic strengths and time delay parameters are varied. $\mathrm {CV}$ is an important statistical measure based on the time intervals between spikes (Pikovsky and Kurths 1997; Masoliver et al. 2017) and which is related to the timing precision of information processing in neural systems (Pei et al. 1996). When $\mathrm {CV}=0$, the neural system exhibits a deterministic periodic spiking, a value that we cannot reach in our model due to the presence of noise. In the double limit $(\sigma _{p,i},\varepsilon ) \rightarrow (0,0)$, the coherence of the spiking due to SISR increases as $\mathrm {CV}\rightarrow 0$, i.e., as $\big [\frac{\sigma _{p,i}^2}{2}\ln (\varepsilon ^{-1})\big ]$ tends to the mid-point of the interval $\big [\varDelta U^l_i(w_e), F_p(\cdot )\big ]$ as the parameter values change. When $\mathrm {CV}=1$, we only have occasional (rare) spiking, leading to a Poissonian distribution of spiking events which are irregular. When $\mathrm {CV}>1$, we have an occurrence of spikes which is even more irregular than that in a spike train with the Poissonian distribution. In these cases (i.e., when $\mathrm {CV}\ge 1$), the quantity $\big [\frac{\sigma _{p,i}^2}{2}\ln (\varepsilon ^{-1})\big ]$ either lies within the interval $\big [\varDelta U^l_i(w_e), F_p(\cdot )\big ]$ but very close to its boundaries or outside the interval, especially when $\mathrm {CV}>1$.

We numerically integrated the Eq. (1) with a step size of $dt=0.008$ for a very long total integration time of $T=3\times 10^{5}$. The integration was performed with the second order Runge-Kutta scheme for Itô stochastic differential equations (Rößler 2009) using the *itoSRI2* method from the Python package *sdeint*. Moreover, each point on the CV curves was obtained after 6 realizations of each of these noise intensities.

The $\mathrm {CV}$ of *N* coupled neurons is defined as (Masoliver et al. 2017):

14 $$\begin{aligned} \mathrm {CV}=\dfrac{\sqrt{\langle \overline{\mathrm {ISI}^2}\rangle -\langle \overline{\mathrm {ISI}}\rangle ^2}}{\langle \overline{\mathrm {ISI}}\rangle }, \end{aligned}$$

where

15 $$\begin{aligned} \begin{aligned} \left\{ \begin{array}{lcl} \langle \overline{\mathrm {ISI}}\rangle &{}=&{}\frac{1}{N}\sum \limits _{i=1}^N \langle \mathrm {ISI}_i \rangle ,\\ \langle \overline{\mathrm {ISI}^2}\rangle &{}=&{}\frac{1}{N}\sum \limits _{i=1}^N \langle \mathrm {ISI}_i^2 \rangle , \end{array}\right. \end{aligned} \end{aligned}$$

in which $\langle \mathrm {ISI}_i \rangle$ and $\langle \mathrm {ISI}_i^2 \rangle$ are the mean and the mean squared (over the total time of simulation *T*) inter-spike intervals of the *i*th neuron, respectively. While $\langle \overline{\mathrm {ISI}}\rangle$ and $\langle \overline{\mathrm {ISI}^2}\rangle$ are the mean (over the total number of neurons *N*) of $\langle \mathrm {ISI}_i \rangle$ and $\langle \mathrm {ISI}_i^2 \rangle$, respectively. The threshold value of the membrane potential variable above which a spike is considered to occur is $v_{\mathrm {th}}=0.0$.

## Simulation results and discussion

### SISR in a single neuron without autapse

In this subsection, we investigate the degree of SISR in a single isolated ML neuron without autapses in excitable regime (i.e., $v_l=1.515<v_{\mathbb {H}}$) and how it varies with the time scale separation parameter $\varepsilon$ and the noise intensity $\sigma _{1,1}=\sigma$. From Eq. (1) and Eqs. (8)–(12), we respectively obtain a single isolated neuron without autapses and the corresponding set of necessary conditions for the occurrence of SISR by setting all coupling strengths to zero, i.e., $\kappa ^a_e=\kappa ^a_c=\kappa _e=\kappa _c=\kappa ^m_e=\kappa ^m_c=0$.

**Fig. 3:**
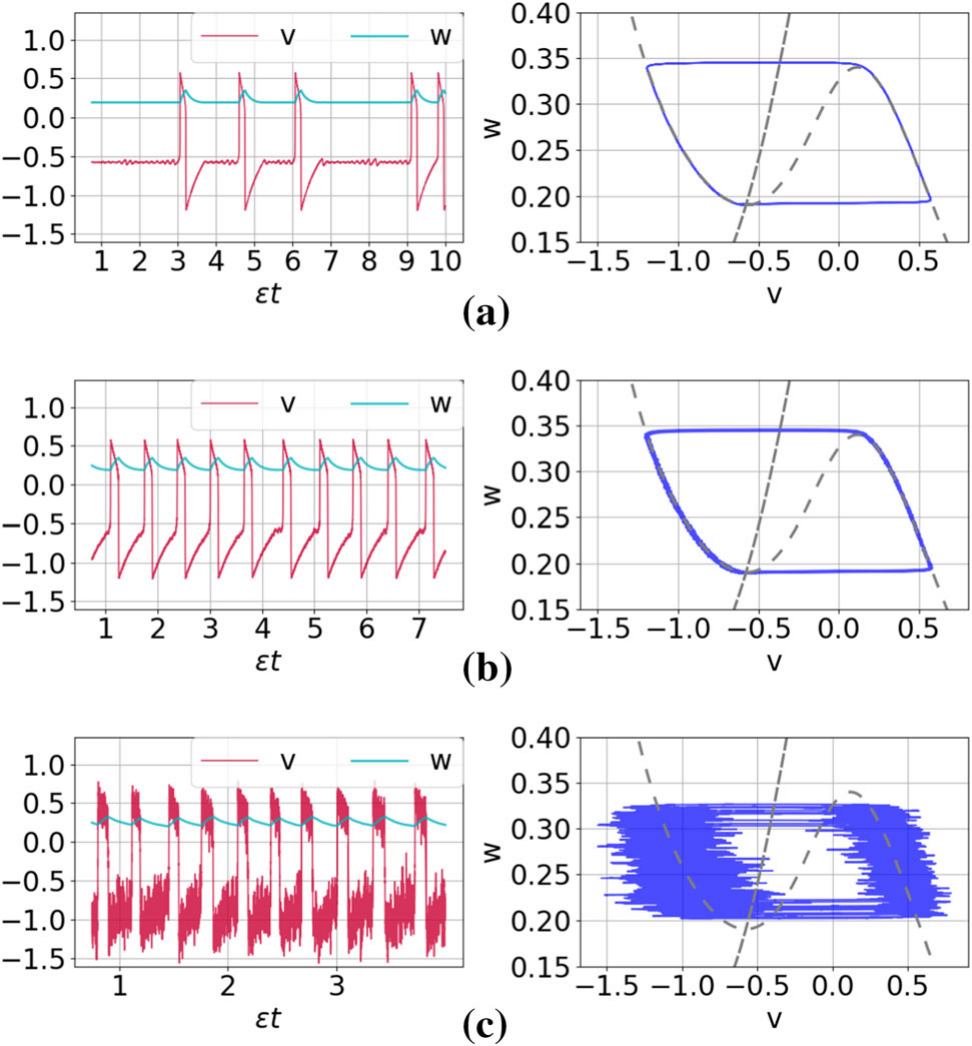
Time series (left) and corresponding phase portraits (right) showing noise-induced coherent oscillations in a single isolated ML neuron in the excitable regime (i.e., $v_l=1.515<v_{\mathbb {H}}$) with $\varepsilon =0.00005$ and for different noise amplitudes: $\sigma =0.0006$, 0.005, and 0.12 in panels **a**–**c**, respectively

In Fig. 3a–c, we show sample trajectories in time series and the corresponding phase portrait for increasing noise intensities at $\varepsilon =0.00005$. We observe that as the noise increases, but within the weak limit (i.e., $\sigma \ll 1$), the coherence of the spikes is not significantly changed.

Figure5 shows the variation of $\mathrm {CV}$ against the noise intensity $\sigma \ll 1$ for several values of the time scale separation parameter $\varepsilon \ll 1$. First, we observe that the smaller $\varepsilon$ is, the larger the interval of the noise amplitude $\sigma$ in which the $\mathrm {CV}$ values are the lowest, i.e., typically below 0.2. This is quite remarkable because in these larger intervals of noise where the $\mathrm {CV}$ values are low, one can actually vary the noise intensity without changing the high degree of coherence of the spiking activity due to SISR. On the other hand, the larger $\varepsilon$ is, the higher the minimum value of $\mathrm {CV}$. Thus, as the conditions in Eq. (8) predict, a high degree of SISR depends on the interplay between the time scale separation parameter and noise intensity in their weak limits $(\sigma ,\varepsilon )\rightarrow (0,0)$.

Returning to Eq. (13), we provide a theoretical explanation (based on the expressions of $\sigma _{min}$ and $\sigma _{max}$) to the fact that the left branch of the CV curve in Fig. 5 is shifted to the right as $\varepsilon$ increases and while the right branch does not significantly move. Furthermore, we use these theoretical expressions in Eq. (13) to accurately calculate the order of magnitude of $\sigma _{min}$ and $\sigma _{max}$ for a single isolated neuron at a given $\varepsilon$. We remind that the explanations and calculations given here for the case of a single isolated neuron also applies to the the rest of the cases investigated in this paper. But as a test-of-principle and for the sake of simplicity, we only show the details for the isolated neuron without autapses.

**Fig. 4:**
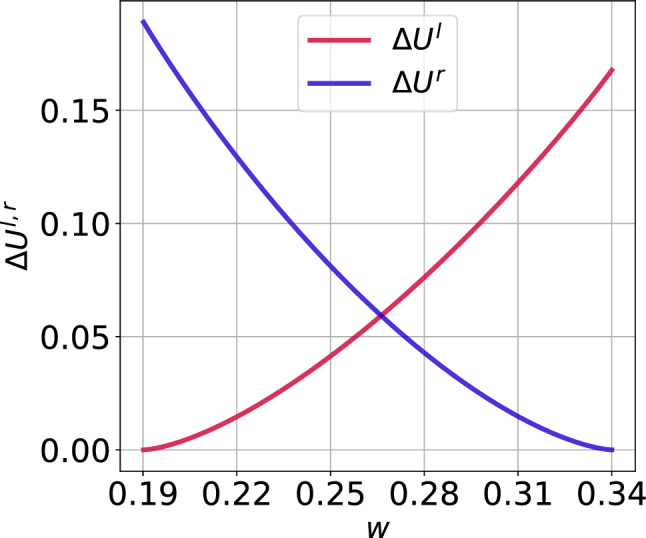
Energy barriers $\varDelta U^{l,r}$ of the single isolated ML neuron against the slow variable *w* in the excitable regime (i.e., $v_l=1.515<v_{\mathbb {H}}$ with $\varepsilon =0.0005$)

**Fig. 5:**
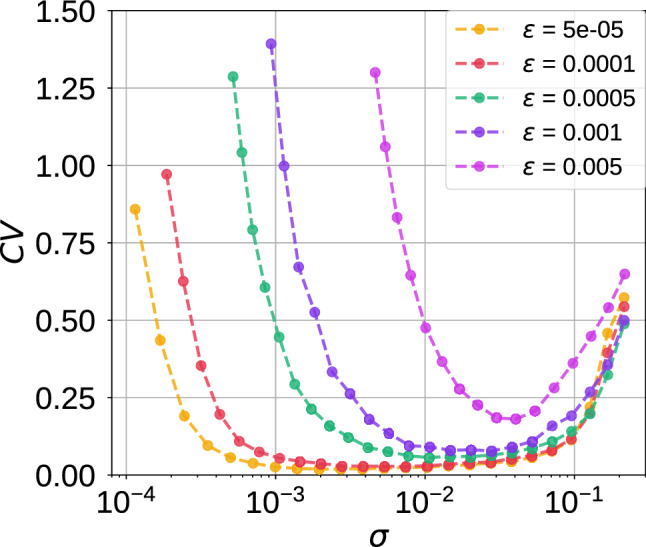
Coefficient of variation $\mathrm {CV}$ against noise amplitude $\sigma$ in a single isolated ML neuron in the excitable regime (i.e., $v_l=1.515<v_{\mathbb {H}}$) for different values of the time scale parameter $\varepsilon$. The intervals of the weak noise intensity in which $\mathrm {CV}<0.2$ shrinks with increasing $\varepsilon$. Furthermore, the minimum $\mathrm {CV}$ values in order of increasing $\varepsilon$ are as follows: $\mathrm {CV}_{\mathrm {min}}= 0.018,~0.028,~0.056,~0.079,~0.18$

Why will the CV curves in Fig. 5 be shifted to the right as $\varepsilon$ increases? To answer this question, we note that for a fixed set of parameter values, $\varDelta U^l(w_e)$ and $F(\cdot )$ in the expressions of $\sigma _{min}$ and $\sigma _{max}$ are also fixed. We further observe that as $\varepsilon$ increases, $\ln (\varepsilon ^{-1})$ decreases, and hence $\sigma _{min}$ increases (since $\varDelta U^l(w_e)$ is fixed), which, therefore, shifts the left branch of the CV curve to be right as $\varepsilon$ increases. On the other boundary, why will the CV curves in Fig. 5 remain almost unchanged as $\varepsilon$ increases? To answer this question, we have to calculate $F(\cdot )$ for the isolated neuron. Figure 4 shows the graph of the left $\varDelta U^l(w)$ and right $\varDelta U^r(w)$ energy barriers given by Eq. (11).

We observe that from the definition of $F(\cdot )$ in Eq. (10) and Fig. 4 that $\varDelta U^l(w)=\varDelta U^r(w)$ at $w=0.2662$ and hence $F(.)=0.059274$ for $\varepsilon =0.0005$. This gives a maximum noise $\sigma _{max} = 1.249\times 10^{-1}$. With the stable unique fixed point evaluated at $(v_e,w_e)=(-0.5767, 0.19019)$, we calculate left energy barrier at $\varDelta U^l(w_e)=1.45\times 10^{-6}$ and hence the corresponding minimum noise of $\sigma _{min} = 6.0\times 10^{-4}$. Comparing $\sigma _{min} = 6.0\times 10^{-4}$ and $\sigma _{max} = 1.249\times 10^{-1}$ with extreme values $\sigma$ in Fig. 5 when $\varepsilon =0.0005$, we observe that the theoretical results of Eq. (13) predict the correct order of magnitude of $\sigma _{min}$ and $\sigma _{max}$. Furthermore, we notice from Fig. 4 that the value of $F(.)=0.059274$ (i.e., the value of $\varDelta U(w)$ when $\varDelta U^l(w)=\varDelta U^r(w)$) does not change as *w* varies. This is why the right branches of the CV curves do not change significantly (i.e., they all have the same order of magnitude) as $\varepsilon$ changes.

In the rest of our numerical simulations, we fix the time scale parameter at $\varepsilon =0.0005$. This very small value is chosen for two reasons: (1) The behavior of SISR at a very small value of $\varepsilon$ is qualitatively the same as at relatively larger values (which maybe biologically more relevant), provided that the interplay between the noise amplitude $\sigma$ and $\varepsilon$ is satisfied according to Eq. (8). (2) The phenomenon of SISR is very pronounced at very small values of $\varepsilon$ (if, of course, Eq. (8) is satisfied), making it easier to understand its behavior.

### SISR in a single neuron with an electrical autapse

In this subsection, we investigate the degree of SISR in a single isolated ML neuron with (only) an electrical autapse in the excitable regime (i.e., $v_l=1.515<v_{\mathbb {H}}$) and how it varies with the autaptic coupling strength $\kappa ^a_e$, time delay $\tau ^a_e$, and the noise intensity $\sigma _{1,1}=\sigma$. In this case, in Eq. (1) and Eqs. (8)–(12), we set $\kappa ^a_c=\kappa _e=\kappa _c=\kappa ^m_e=\kappa ^m_c=0$, except $\kappa ^a_e\ne 0$.

As we pointed out earlier, time-delayed couplings may invoke a saddle-node onto limit cycles (SNLC) bifurcation, leading to the emergence of self-sustained spiking activity in the autaptic neuron even in the absence of noise (Schöll et al. 2009). SNLC may occur even if the Hopf bifurcation parameter is fixed in the excitable regime (i.e., $v_l=1.515<v_{\mathbb {H}}$) identified in Fig. 2a. Thus, it is indispensable to identify and avoid time-delayed coupling values leading to SNLC in the zero-noise dynamics.

**Fig. 6:**
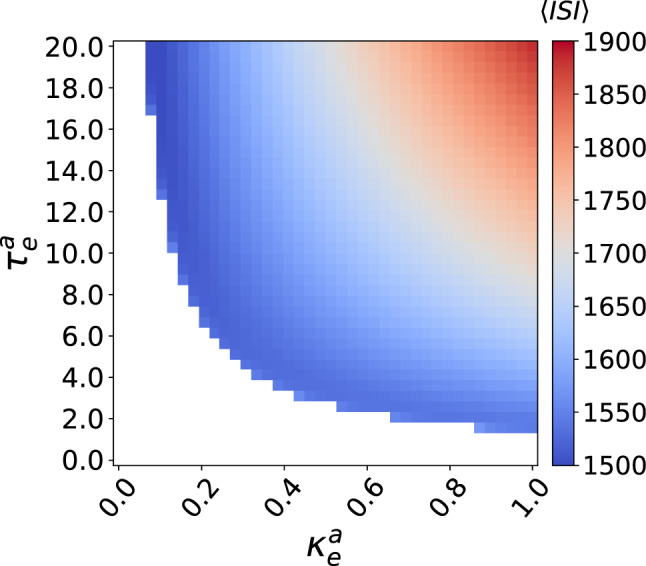
The mean inter-spike interval $\langle \mathrm {ISI} \rangle$ is color coded in the $(\kappa ^a_e - \tau ^a_e)$ plane for the deterministic (i.e., $\sigma _{1,1}=\sigma =0$) ML neuron with an eletrical autapse. The white region represents the excitable regime (i.e., predisposition for SISR) and the colored regions represent the oscillatory regime (i.e., undesired regime) invoked by autaptic time-delayed couplings via SNLC bifurcations. $v_l=1.515<v_{\mathbb {H}}$, $\varepsilon =0.0005$

Figure 6 shows a color coded $\mathrm {ISI}$ in a two-parameter ($k^a_e$, $\tau ^a_e$) deterministic bifurcation diagram. The white region represents the desired excitable regime (where no spike occurs and thus no $\mathrm {ISI}$), while the colored regions represent the undesired oscillatory regime (with non-zero $\mathrm {ISI}$) induced by SNCL.

**Fig. 7:**
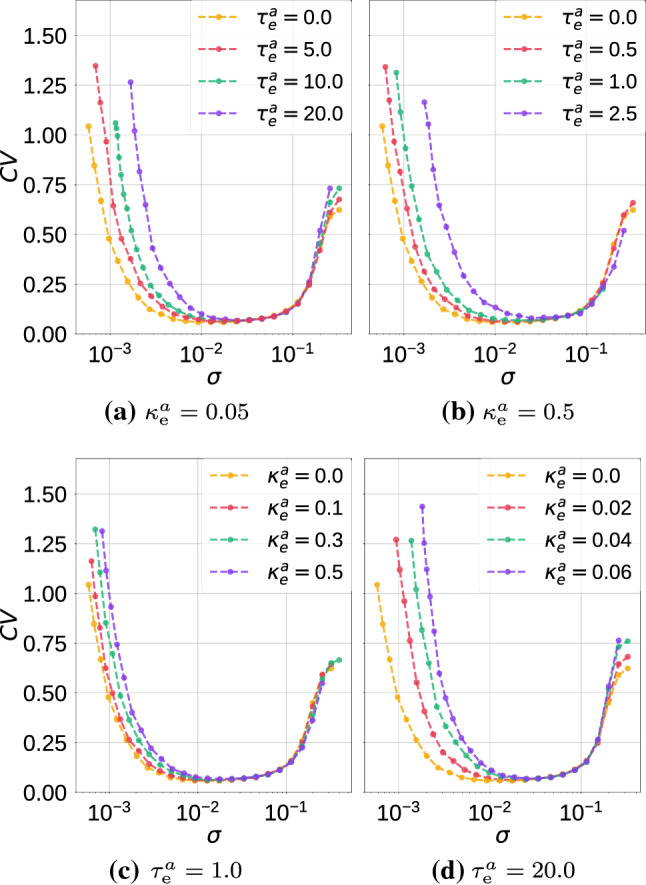
Coefficient of variation $\mathrm {CV}$ against noise amplitude $\sigma$ for parameter combinations of the electrical autapse $(\kappa ^a_{\mathrm {e}},\tau ^a_{\mathrm {e}})$ in a single isolated ML neuron. We observe variations in the electrical autaptic parameters do not significantly affect the high degree of SISR, but they can shrink the interval of the noise amplitude in which this degree remains high, by shifting the left branch of the $\mathrm {CV}$ curves to relatively larger noise intensities. $v_l=1.515<v_{\mathbb {H}}$, $\varepsilon =0.0005$

In Fig. 7, we show the variation of $\mathrm {CV}$ against the noise amplitude $\sigma$ with values of $\kappa ^a_e$ and $\tau ^a_e$ taken from the excitable regime in Fig. 6. In Fig. 7a, we choose a weak autaptic coupling $\kappa ^a_e=0.05$ and vary the time delay $\tau ^a_e\in \{0.0, 5.0, 10.0, 20.0\}$. We observe that in this weak autaptic coupling regime, the time delay has no effect on the high degree of SISR achieved, as all the $\mathrm {CV}$ curves remain at almost the same (low) value. However, as the time delay $\tau ^a_e$ becomes longer, the intervals of $\sigma$ in which the $\mathrm {CV}$ curves are the lowest shrink as the left branch of the $\mathrm {CV}$ curves are shifted to the right, i.e., to relatively larger noise intensities.

In Fig. 7b, where the autaptic strength becomes stronger, i.e., $\kappa ^a_e=0.5$, the $\mathrm {CV}$ curves qualitatively behaves as in Fig. 7a, except that the degree of SISR become very sensitive to small changes in length of time delays: we notice in Fig. 7b, $\tau ^a_e$ varies only between 0.0 and 2.5 in order to have the qualitative behavior in Fig. 7a.

In Fig. 7c and d, we now fixed $\tau ^a_e$ at a short (e.g., $\tau ^a_e=1.0$) and long (e.g., $\tau ^a_e=20.0$) time delays, respectively, and vary the autaptic coupling strength. We observe that the minimum values of the $\mathrm {CV}$ curves are not significantly changed as the $\kappa ^a_e$ changes. Nevertheless, the intervals of $\sigma$ in which the $\mathrm {CV}$ values are the lowest, shrink as the left branches of the $\mathrm {CV}$ curves are again shifted to the right. Moreover, in long time delay regimes such as in Fig. 7d, the degree of SISR becomes very sensitive to variations of the autaptic coupling strength. Here, $\kappa ^a_e$ varies only up to 0.06 in order to have the same qualitative behavior observed in Fig. 7c where $\kappa ^a_e$ varies up to 20.0.

### SISR in a single neuron with a chemical autapse

In this subsection, we investigate the degree of SISR in a single isolated ML neuron with (only) an inhibitory chemical autapse and how it varies with the autaptic coupling strength $\kappa ^a_{\mathrm {c,inh}}$, time delay $\tau ^a_{\mathrm {c,inh}}$, and the noise intensity $\sigma$. In this case, in Eq. (1) and Eqs. (8)–(12), we set $\kappa ^a_e=\kappa _e=\kappa _c=\kappa ^m_e=\kappa ^m_c=0$, except $\kappa ^a_c\ne 0$. It should be noted that we do not consider an excitatory chemical autapse in this case. This is because with this type of autapse, the deterministic ML neuron is always in the oscillatory regime—the undesired predisposition for SISR. For the whole range of parameter values of the inhibitory chemical autapse used, the isolated ML neuron always remains excitable.

**Fig. 8:**
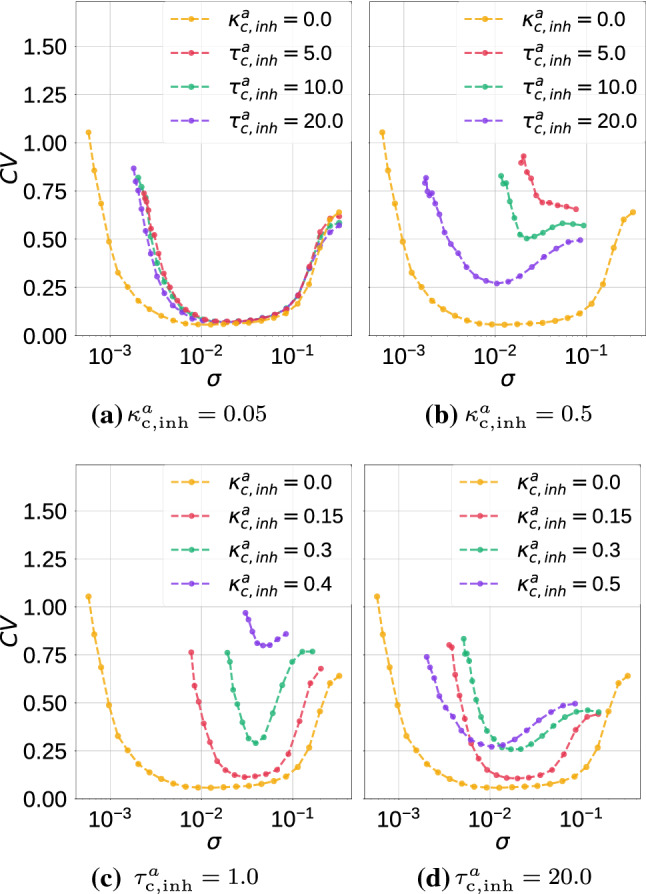
Coefficient of variation $\mathrm {CV}$ against noise amplitude $\sigma$ for parameter combinations of the inhibitory chemical autapse $(\kappa ^a_{\mathrm {c,inh}},\tau ^a_{\mathrm {c,inh}})$ in a single isolated ML neuron. We observe that variations in these parameters significantly affect the degree of SISR by shifting the entire $\mathrm {CV}$ curve to higher values and by shrinking, on both ends, the interval of the noise amplitude in which this degree remains relatively high. Longer autaptic time delays enhance SISR, while stronger autaptic couplings destroy SISR. $v_l=1.515<v_{\mathbb {H}}$, $\varepsilon =0.0005$

In Fig. 8, we show the variation of $\mathrm {CV}$ against the noise amplitude $\sigma$ with values of $\kappa ^a_{\mathrm {c,inh}}$ and $\tau ^a_{\mathrm {c,inh}}$. In Fig. 8a, at a weak autaptic coupling $\kappa ^a_{\mathrm {c,inh}}=0.05$, we vary the time delay $\tau ^a_{\mathrm {c,inh}}\in \{0.0, 5.0, 10.0, 20.0\}$. In this case, we observe that variations in the time delay have no effect on the high degree of SISR achieved. All the $\mathrm {CV}$ curves remain at almost the same (low) value.

In Fig. 8b, where the autaptic strength becomes stronger, i.e., $\kappa ^a_{\mathrm {c,inh}}=0.5$, the $\mathrm {CV}$ curves change significantly, both qualitatively and quantitatively. Here, as the time delay increases from a non-zero value, the minimum of the $\mathrm {CV}$ curves get lower, indicating a higher degree of SISR.

In Fig. 8c and d, we fix the autaptic time delays at $\tau ^a_{\mathrm {c,inh}}=1.0$ and $\tau ^a_{\mathrm {c,inh}}=20.0$, respectively, and vary the autaptic strength $\kappa ^a_{\mathrm {c,inh}}\in \{0.0, 0.15, 0.3, 0.4, 0.5\}$. We observe that: $(\mathrm {i})$ SISR is very sensitive to small variations in the autaptic coupling strength $\kappa ^a_{\mathrm {c,inh}}$ and $(\mathrm {ii})$ the time delay $\tau ^a_{\mathrm {c,inh}}$ and the coupling strength $\kappa ^a_{\mathrm {c,inh}}$ have opposite effects on the degree of SISR. While larger values of $\tau ^a_{\mathrm {c,inh}}$ increase the degree of SISR, larger values of $\kappa ^a_{\mathrm {c,inh}}$ decrease it.

Furthermore, the deterioration of SISR with an inhibitory chemical autapse manifests in two ways: $(\mathrm {i})$ higher $\mathrm {CV}$ curves and hence lower degree of SISR and $(\mathrm {ii})$ smaller intervals of the noise intensity in which the degree of SISR is relatively high—notice that the shrinking of the noise interval happens on both the left and the right branch of the $\mathrm {CV}$ curves. Recall from the previous subsection that the deterioration of SISR with an electrical autapse consisted only in a reduction of this interval and also, only from the left branch of the $\mathrm {CV}$ curves.

### SISR in a single motif network

In this subsection, we investigate the degree of SISR in a single isolated motif without autapses and with either only electrical synapses or only chemical synapses between the three neurons and how it varies with the synaptic time-delayed couplings ($\kappa _e$, $\tau _e$) or ($\kappa _c$, $\tau _c$), and the noise intensity $\sigma _{1,1}=\sigma _{1,2}=\sigma _{1,3}=\sigma$. In motifs with electrical synapses, i.e., the topologies in Fig. 1b, we set in Eq. (1) and Eqs. (8)–(12), $\kappa ^a_e=\kappa ^a_c=\kappa _c=\kappa ^m_e=\kappa ^m_c=0$, except $\kappa _e\ne 0$. In the same fashion, for motifs with chemical synapses, i.e., the topologies in Fig. 1c and d, we set $\kappa ^a_e=\kappa ^a_c=\kappa _e=\kappa ^m_e=\kappa ^m_c=0$, except $\kappa _c\ne 0$ in Eq. (1) and Eqs. (8)–(12).

**Fig. 9:**
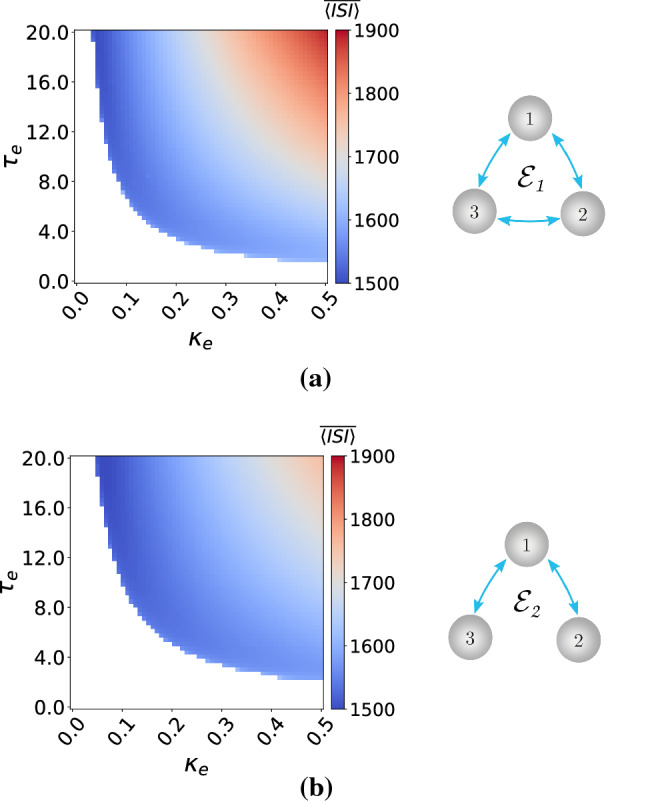
Excitability maps (left) and corresponding topology (right) of electrically coupled motif layer networks. $\overline{\langle \mathrm {ISI}\rangle }$ is color coded with the white region representing the excitable regime and the colored regions the oscillatory regimes. $v_l=1.515<v_{\mathbb {H}}$, $\varepsilon =0.0005$

**Fig. 10:**
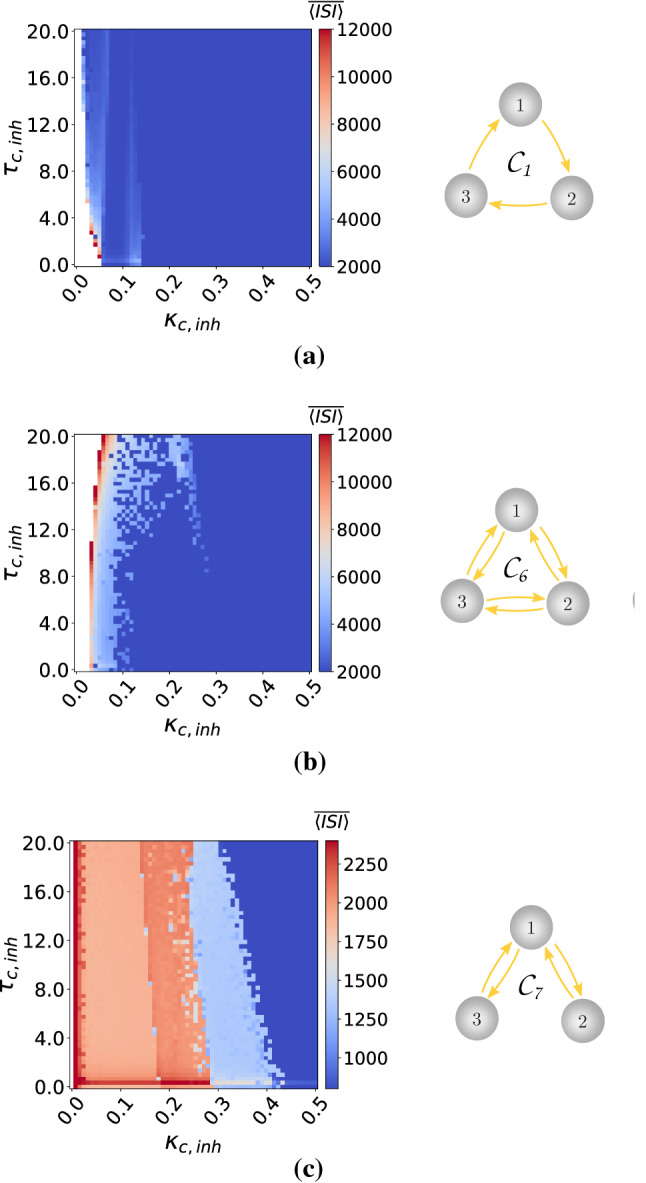
Excitability maps (left) and corresponding topology (right) of motif layer networks with inhibitory chemical synapses. $\langle \overline{\mathrm {ISI}}\rangle$ is color coded with the white region representing the excitable regime and the colored regions the oscillatory regimes. Notice that $\mathcal {C}_7$ does not admit excitability. $v_l=1.515<v_{\mathbb {H}}$, $\varepsilon =0.0005$

To guarantee the excitability of each of these motifs (where we fix $\varepsilon =0.0005$ and $v_l=1.515$ in each neuron), we compute two-parameter deterministic bifurcation diagrams with respect to the synaptic parameters ($\kappa _e$, $\tau _e$) or ($\kappa _c$, $\tau _c$). Simulations indicate that electrical and inhibitory chemical synapses can set the deterministic motifs into either an excitable or an oscillatory regime, depending on the values of the synaptic coupling strengths ($\kappa _e,\kappa _{\mathrm {c,inh}}\in [0.0,0.5]$) and time delays ($\tau _e, \tau _{\mathrm {c,inh}}\in [0.0,20.0]$), see Figs.9 and 10. On the other hand, all the parameter values of the excitatory chemical synapses (figures not shown) set the deterministic motifs into the oscillatory regime. Hence, we do not investigate SISR in motifs layer networks with excitatory chemical synapses.

Our simulations have also indicated that (figures not shown) the motifs $\mathcal {C}_2$–$\mathcal {C}_5$, with only chemical inhibitory synapses, are always in the excitable regime for all the synaptic parameter values used. Moreover, Fig. 10c indicates that the $\mathcal {C}_7$ topology with chemical inhibitory synapses does not admit excitability. Hence, we also exclude the $\mathcal {C}_7$ topology from our investigations of SISR.

**Fig. 11:**
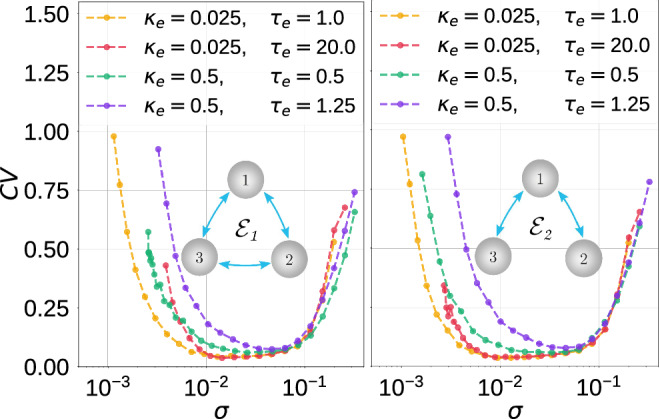
Coefficient of variation $\mathrm {CV}$ against noise amplitude $\sigma$ for parameter combinations $(\kappa _{\mathrm {e}},\tau _{\mathrm {e}})$ in the electrical motifs topology indicated. $v_l=1.515<v_{\mathbb {H}}$, $\varepsilon =0.0005$

**Fig. 12:**
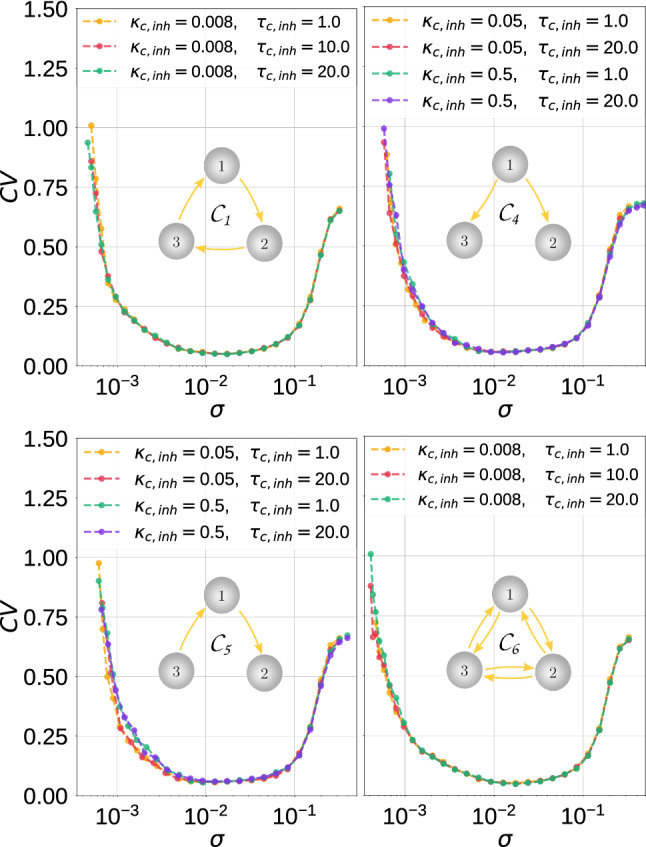
Coefficient of variation $\mathrm {CV}$ against noise amplitude $\sigma$ for parameter combinations $(\kappa _{\mathrm {c,inh}},\tau _{\mathrm {c,inh}})$ in the inhibitory chemical motifs topology indicated. In these motifs, SISR is more robust to parametric changes than in the motifs considered in Fig. 13. $v_l=1.515<v_{\mathbb {H}}$, $\varepsilon =0.0005$

**Fig. 13:**
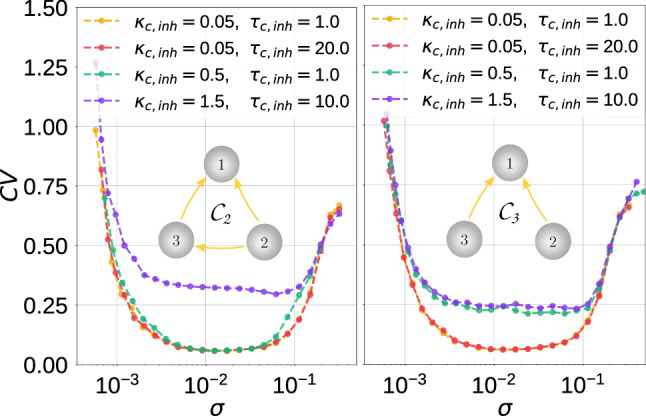
Coefficient of variation $\mathrm {CV}$ against noise amplitude $\sigma$ for parameter combinations $(\kappa _{\mathrm {c,inh}},\tau _{\mathrm {c,inh}})$ in the inhibitory chemical motifs topology indicated. In these motifs the degree is SISR can be significantly deteriorated (see the purple curves in each panel) as opposed to the degree in the motifs in $v_l=1.515<v_{\mathbb {H}}$, $\varepsilon =0.0005$

In Figs. 11, 12 and 13, we present the variations of the $\mathrm {CV}$ curves against noise intensity for the indicated motif topology and different intra-motif time-delayed coupling parameter values.

In Fig. 11, with the electrical motifs $\mathcal {E}_1$ and $\mathcal {E}_2$, respectively, we observe that weaker coupling strengths $\kappa _{\mathrm {e}}$ and shorter time delays $\tau _e$ lead to a higher degree of SISR, especially at weaker noise intensities. There are no significant differences in the degree of SISR in $\mathcal {E}_1$ and $\mathcal {E}_2$.

In Fig. 12, with the inhibitory chemical motifs $\mathcal {C}_1$, $\mathcal {C}_4$, $\mathcal {C}_5$, and $\mathcal {C}_6$, we mainly observe that the high degree of SISR achieved in the corresponding motifs are quite robust to parametric changes in the synapses—the $\mathrm {CV}$ curves remain very low and the intervals of the noise intensity in which these high degrees are achieved remain unchanged as the parameters change.

In Fig. 13, the $\mathrm {CV}$ curves of motifs $\mathcal {C}_2$ and $\mathcal {C}_3$ show significant fluctuations in the degree of SISR as the parameters change. In these two cases, stronger coupling strengths and longer time delays lead to a lower degree of SISR. See, e.g., the purple $\mathrm {CV}$ curves in panels of Fig. 13, where $\kappa _{\mathrm {c,inh}}=1.5$ and $\tau _{\mathrm {c,inh}}=10.0$. We observe that these curves are shifted upwards to higher $\mathrm {CV}$ values compared to other curves, thus reducing the degree of SISR. The minimum $\mathrm {CV}$ values of these purple curves are: $\mathrm {CV}_{min}=0.293$ in the left panel and $\mathrm {CV}_{min}=0.232$ in the right panel of Fig. 13.

Comparing the degree of SISR in isolated neurons and motif networks, we can conclude that the degree of SISR in motifs can be as good as in the isolated neuron, but not better. As we can see from Figs. 11, 12 and 13, as the strength of synaptic couplings increases, the interval of the noise amplitude within which the degree of SISR is high decreases and the minimum of the CV curves rises to a small but significant value. But as the strength of these synaptic couplings decreases, this interval of noise increases and the minimum of the CV descends to lower values. Eventually, when the synaptic couplings decrease to vanishingly small values (e.g., $\kappa _{c,inh}=0.008$) or even to zero (in which case we have isolated neurons) the degree of SISR in the motifs becomes very close to the degree of SISR in the isolated neuron and identical when the synaptic coupling becomes zero. Therefore, we conclude that the motif can at most do as well as an isolated neuron, but not better.

A natural and interesting question to investigate is whether this significantly poor degree of SISR can be enhanced in the motifs $\mathcal {C}_2$ and $\mathcal {C}_3$. In the sequel, we present two enhancement strategies for SISR in these motifs.

### Autaptic enhancement of SISR in single motifs

In this subsection, we present one enhancement strategy of SISR in single motifs that is based on the use of autapses. To illustrate the efficacy of this strategy, we use the motifs $\mathcal {C}_2$ and $\mathcal {C}_3$ from Fig. 13, with time-delayed coupling fixed at $\kappa _{\mathrm {c,inh}}=1.5$ and $\tau _{\mathrm {c,inh}}=10.0$. With this setting and in the absence of autapses, the degree of SISR is relatively poor, i.e., $\mathrm {CV}_{min}=0.293$ in the motif $\mathcal {C}_2$ and $\mathrm {CV}_{min}=0.232$ in the motif $\mathcal {C}_3$ as indicated by the purple curves in the panels of Fig. 13.

**Fig. 14:**
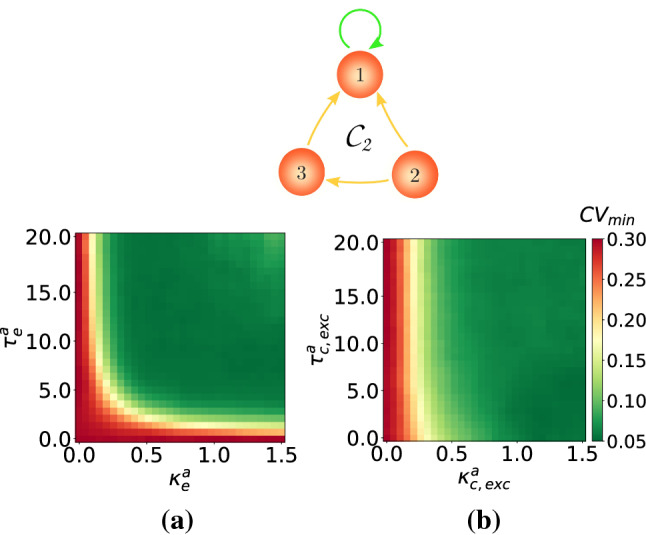
Minimum coefficient of variation $\mathrm {CV}_{\mathrm {min}}$ for the motif $\mathcal {C}_2$ against: **a** electrical autaptic parameters $(\kappa ^a_e,\tau ^a_e)$ and **b** excitatory chemical autaptic parameters $(\kappa ^a_{\mathrm {c,exc}},\tau ^a_{\mathrm {c,exc}})$. There is an enhancement in the degree of SISR from $\mathrm {CV}_{\mathrm {min}}=0.293$ in the absence of autapses to $\mathrm {CV}_{\mathrm {min}}\approx 0.06$ in their presence. $\kappa _{\mathrm {c,inh}}=1.5$, $\tau _{\mathrm {c,inh}}=10.0$, $v_l=1.515<v_{\mathbb {H}}$, $\varepsilon =0.0005$

**Fig. 15:**
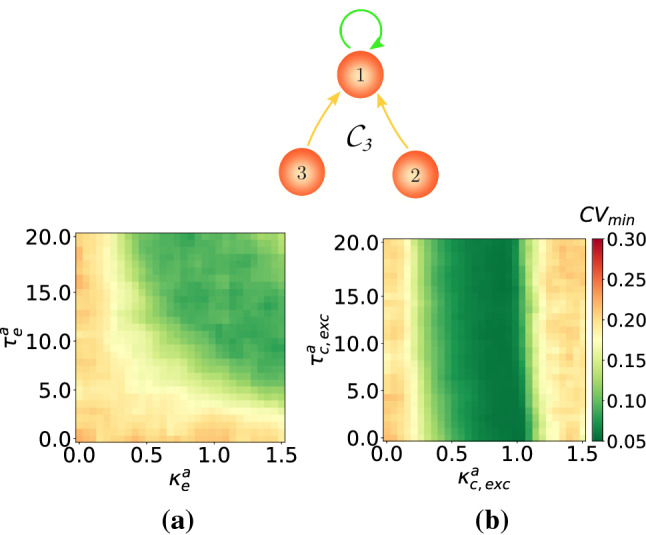
Minimum coefficient of variation $\mathrm {CV}_{\mathrm {min}}$ for the motif $\mathcal {C}_3$ against: **a** electrical autaptic parameters $(\kappa ^a_e,\tau ^a_e)$ and **b** excitatory chemical autaptic parameters $(\kappa ^a_{\mathrm {c,exc}},\tau ^a_{\mathrm {c,exc}})$. There is an enhancement in the degree of SISR from $\mathrm {CV}_{\mathrm {min}}=0.232$ in the absence of autapses to $\mathrm {CV}_{\mathrm {min}}\approx 0.09$ and $\mathrm {CV}_{\mathrm {min}}\approx 0.06$ in **a** and **b**, respectively, in the presence of autapses. $\kappa _{\mathrm {c,inh}}=1.5$, $\tau _{\mathrm {c,inh}}=10.0$, $v_l=1.515<v_{\mathbb {H}}$, $\varepsilon =0.0005$

The goal of this strategy is to lower the value of these $\mathrm {CV}_{\mathrm {min}}$ using an autapse with appropriate parameter values. Our simulations have indicated (not shown) that an inhibitory chemical autapse is not effective with this strategy. The reason is that this type of autapse puts the motifs into the (undesired) oscillatory regime in the absence of noise. Inserting an electrical autapse (with $\kappa ^a_{\mathrm {e}}\in [0.0,1.5]$, $\tau ^a_{\mathrm {e}}\in [0.0,20.0]$) or an excitatory chemical autapse (with $\kappa ^a_{\mathrm {c,exc}}\in [0.0,1.5]$, $\tau ^a_{\mathrm {c,exc}}\in [0.0,20.0]$) on neuron number one of these motifs, kept the motifs in the (desired) excitable regime. Furthermore, our extensive numerical investigations have indicated that this enhancement strategy is most efficient when the electrical autapse or the excitatory chemical autapse is attached only to the neuron with the highest in-degree, i.e., the neuron number one of the motifs. Thus, we only show the results of this case in Fig. 14 for motif $\mathcal {C}_2$ and in Fig. 15 for motif $\mathcal {C}_3$. It is worth noting that this result on the enhancement of SISR by an autapse attached to the neuron with the highest in-degree may not be generally robust and may not apply to other types of motif or larger neural networks not considered in this study.

Figure 14a and b, respectively, show the color coded variation of the $\mathrm {CV}_{\mathrm {min}}$ of the motif $\mathcal {C}_2$ with respect to the time-delayed electrical or excitatory chemical autaptic couplings (represented by the green self-feedback loop) attached to neuron number one of the motif. We observe that these autapses can significantly improve the degree of SISR in the motif $\mathcal {C}_2$. But, in the presence autapses, $\mathcal {C}_2$ with $\kappa _{\mathrm {c,inh}}=1.5$ and $\tau _{\mathrm {c,inh}}=10.0$ has a very low $\mathrm {CV}_{\mathrm {min}}\approx 0.06$, i.e., a much higher degree of SISR. We further notice that the excitatory chemical autapse outperforms the electrical autapse in this enhancement strategy. That is, the former autapse provides a larger range of parameters values in which a high degree of SISR is achieved than the latter, especially at stronger couplings and shorter time delays.

In Fig. 15**a** and **b**, respectively, we show the color coded variation of the $\mathrm {CV}_{\mathrm {min}}$ of the motif $\mathcal {C}_3$ against the time-delayed electrical and excitatory chemical autaptic couplings. In this case, we observe that the degree the of SISR is considerably enhanced, with a drop from $\mathrm {CV}_{\mathrm {min}}=0.232$ to $\mathrm {CV}_{\mathrm {min}}\approx 0.09$ for an electrical autapse and $\mathrm {CV}_{\mathrm {min}}\approx 0.06$ for an excitatory chemical autpase. However, in terms of parameter ranges, the electrical autapse is more efficient than the excitatory chemical autapse for stronger couplings $\kappa ^a_e>1.0$ and longer delays $\tau ^a_e>5.0$. The excitatory chemical autapse is better at enhancing SISR for intermediate $0.25<\kappa ^a_{c,exc}<1.0$ coupling strengths and all time delays. Moreover, we can also see that with this autaptic enhancement strategy, the degree of SISR is best enhanced in motif topology $\mathcal {C}_2$ than $\mathcal {C}_3$.

### Enhancement of SISR based on multiplexing

**Fig. 16:**
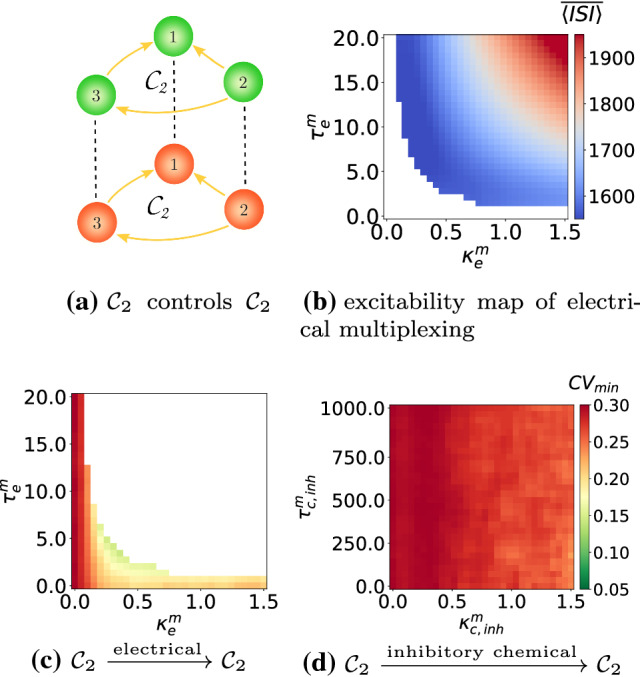
Excitability map in **b** showing $\langle \overline{\mathrm {ISI}}\rangle$ of the multiplexing scheme in **a** with electrical synapses in the absence of noise. $\mathrm {CV}_{\mathrm {min}}$ for the lower (in red) motif layer $\mathcal {C}_2$ against: electrical $(\kappa ^m_e,\tau ^m_e)$ and inhibitory chemical $(\kappa ^m_{\mathrm {c,inh}},\tau ^m_{\mathrm {c,inh}})$ multiplexing parameters in **c** and **d** respectively. There is an enhancement in the degree of SISR from $\mathrm {CV}_{\mathrm {min}}=0.293$ in the absence of multiplexing to $\mathrm {CV}_{\mathrm {min}}\approx 0.12$ and $\mathrm {CV}_{\mathrm {min}}\approx 0.25$ in **c** and **d** in the presence of multiplexing, respectively. $(\kappa _{\mathrm {c,inh}},\tau _{\mathrm {c,inh}})=(1.5,10.0)$ in the lower motif, $(\kappa _{\mathrm {c,inh}},\tau _{\mathrm {c,inh}})=(0.05,20.0)$ in the upper motif. $v_l=1.515<v_{\mathbb {H}}$, $\varepsilon =0.0005$

**Fig. 17:**
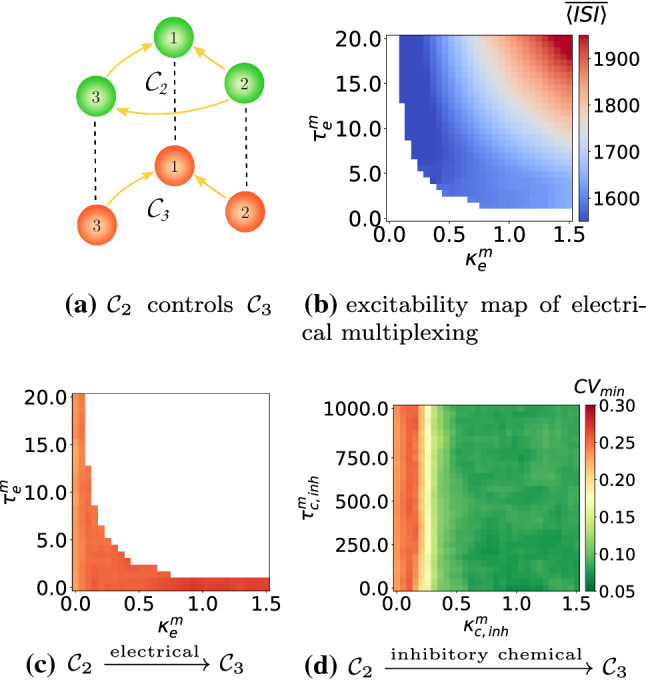
Excitability map **b** showing $\langle \overline{\mathrm {ISI}}\rangle$ of the multiplexing scheme in **a** with electrical synapses in the absence of noise. $\mathrm {CV}_{\mathrm {min}}$ for the motif layer $\mathcal {C}_3$ against: electrical $(\kappa ^m_e,\tau ^m_e)$ and inhibitory chemical $(\kappa ^m_{\mathrm {c,inh}},\tau ^m_{\mathrm {c,inh}})$ multiplexing parameters in **c** and **d**, respectively. There is no enhancement in the degree of SISR in **c** and an enhancement from $\mathrm {CV}_{\mathrm {min}}=0.232$ in the absence of multiplexing to $\mathrm {CV}_{\mathrm {min}}\approx 0.058$ in **d** in the presence of multiplexing. $(\kappa _{\mathrm {c,inh}},\tau _{\mathrm {c,inh}})=(1.5,10.0)$ in the lower motif, $(\kappa _{\mathrm {c,inh}},\tau _{\mathrm {c,inh}})=(0.05,20.0)$ in the upper motif. $v_l=1.515<v_{\mathbb {H}}$, $\varepsilon =0.0005$

In this subsection, we present another enhancement strategy of SISR in a motif, based on multiplexing. That is, we connect two motif layers into a multiplex network, where each neuron in one layer is only connected to the replica neuron in the other layer; see Figs. 16a and 17a. In each of these figures, the upper motif layer in green is such that, in isolation, the degree of SISR is very high, i.e., $\mathrm {CV}_{\mathrm {min}}=0.058$ in Fig. 16a and 17a. The lower motif layer in red is the one with a poor degree of SISR when it is in isolation (i.e., $\mathrm {CV}_{\mathrm {min}}=0.293$ in Fig. 16a and $\mathrm {CV}_{\mathrm {min}}=0.232$ in Fig. 17a), and in which we want to enhance the degree of SISR by connecting it to the upper motif in a multiplexing manner.

In Fig. 16, we represent the efficiency of this enhancement strategy of SISR in the $\mathcal {C}_2$ motif layer in red when it is multiplexed to another $\mathcal {C}_2$ motif layer in green, with electrical inter-motif connections or single unidirectional inhibitory chemical inter-motif connections. Excitatory chemical connections were not used in this strategy because they will induce self-sustained deterministic oscillations. Furthermore, not all time-delayed coupling values of electrical inter-motif connections would set the system into the desired excitable regime. We can see in Fig. 16b the range of values of the inter-motif connection parameters—$\kappa ^m_e$ and $\tau ^m_e$—(the white region) in which the entire multiplex network remains in the excitable regime. Only the excitable values of $\kappa ^m_e$ and $\tau ^m_e$ are used in the enhancement strategy. When we use the $\mathcal {C}_2$-$\mathcal {C}_2$ network with electrical multiplexing, we can identify multiplexing parameters from Fig. 16c that improve the degree of SISR in the red motif from $\mathrm {CV}_{\mathrm {min}}=0.293$ when it is in isolation to $\mathrm {CV}_{\mathrm {min}}=0.12$ when it is multiplexed with itself. However, this enhancement strategy is not as efficient when multiplexing connections are mediated by inhibitory chemical couplings, see Fig. 16d, where the degree of SISR is improved by just a little, i.e., $\mathrm {CV}_{\mathrm {min}}=0.25$.

In Fig. 17c with the $\mathcal {C}_2$-$\mathcal {C}_3$ configuration, we now try to enhance SISR in the $\mathcal {C}_3$ motif. The multiplexing enhancement strategy does not work if the multiplexing is mediated by electrical connections. But in Fig. 17d, when inter-motif couplings are mediated by inhibitory chemical connections, the enhancement strategy becomes very efficient. In this case we have $\mathrm {CV}_{\mathrm {min}}=0.058$ in $\mathcal {C}_3$.

We note that in this multiplexing enhancement strategy of SISR, the motif in which we aim at enhancing SISR (i.e., the red lower motifs in Fig. 16a and 17a which have a relatively poor degree of SISR in isolation) is connected in a multiplexed fashion to another motif in which the degree of SISR is very high in isolation. It is worth pointing out here that during the enhancement of SISR in the red lower motifs in Fig. 16d and 17d (for the time-delayed couplings values indicated), the high degree of SISR in the green upper motifs is not affected, except in the case where we have electrical multiplexing between the motifs. In this case (see, e.g., Fig. 16c), while the degree of SISR is enhanced in the red lower motif (i.e., $\mathrm {CV}_{\mathrm {min}}$ goes from 0.293 when it is in isolation to 0.12 when it is multiplexed to the green upper motif), the high degree of SISR in the green upper motif is significantly deteriorated (i.e., $\mathrm {CV}_{\mathrm {min}}$ goes from 0.058 in isolation to 0.19 when it is multiplexed). Hence, in a $\mathcal {C}_2$-$\mathcal {C}_2$ multiplex network configuration, inhibitory chemical couplings between the motifs is the way to go if we want to enhance SISR in one motif without significantly deteriorating SISR in the other motif.

## Summary and conclusions

In this work, the phenomenon of self-induced stochastic resonance (SISR) in the Morris–Lecar (ML) neuron model was systematically investigated. First, we established the analytical conditions necessary for the occurrence of SISR in a motif layer network of ML neurons. Then, from our extensive numerical simulations, we found that:

- In a single isolated ML neuron without autapses, decreasing the time scale separation parameter $\varepsilon$ between the membrane potential *v* and the ionic current *w* variable leads to $(\mathrm {i})$ a significant increase in the degree of SISR and $(\mathrm {ii})$ an increase in the interval of the noise intensity in which the degree of SISR remains high.
- In a single isolated ML neuron with (only) an electrical autapse, the degree of SISR is not significantly sensitive to variations of the autaptic parameters ($\kappa ^a_e$, $\tau ^a_e$). However, at stronger autaptic coupling strengths, the degree of SISR is relatively more sensitive to small changes in time delays than at weaker coupling strengths. This behavior is also observed with longer and shorter time delays as the coupling strength changes.
- In a single isolated ML neuron with (only) an inhibitory chemical autapse, the degree of SISR and the interval of the noise intensity in which a relatively high degree of SISR can be achieved is found to be very sensitive to changes in the autaptic parameters ($\kappa ^a_{\mathrm {c,inh}}$, $\tau ^a_{inh}$). While stronger autaptic coupling strengths deteriorate SISR, longer autaptic time delays enhances SISR.
- In single-motif layer networks with electrical connections $\mathcal {E}_1$ and $\mathcal {E}_2$, we observed that the weaker coupling strengths $\kappa _{\mathrm {e}}$ and shorter time delays $\tau _e$ are, the higher the degree of SISR, especially at weaker noise intensities. On the other hand, single-motif layer networks with inhibitory chemical connections show different behaviors. In the inhibitory chemical motifs $\mathcal {C}_1$, $\mathcal {C}_4$, $\mathcal {C}_5$, and $\mathcal {C}_6$, the high degrees of SISR are robust to changes in the synaptic parameters as the $\mathrm {CV}$ values remain very low and the intervals of the noise intensity in which these high degrees are achieved remain unchanged. However, SISR in the inhibitory chemical motifs $\mathcal {C}_2$ and $\mathcal {C}_3$ show sensitivity to changes in the coupling strength $\kappa _{\mathrm {c,inh}}$ and time delay $\tau _{\mathrm {c,inh}}$, i.e., the larger $\kappa _{\mathrm {c,inh}}$ and $\tau _{\mathrm {c,inh}}$ are, the more deteriorated is SISR.
- It is shown that the poor degree of SISR in the single-motif layer networks $\mathcal {C}_2$ and $\mathcal {C}_3$ can be enhanced using two different strategies. In the enhancement strategy based on autapses, it was shown that electrical autapses with stronger couplings ($\kappa ^a_{\mathrm {e}}>0.25$) and longer time delays ($\tau ^a_{\mathrm {e}}>5.0$) or excitatory chemical autapses with stronger ($\kappa ^a_{\mathrm {c,exc}}>0.25$) and longer time delays ($\tau ^a_{\mathrm {c,exc}}\ge 0.0$) enhances the degree of SISR in $\mathcal {C}_2$ from $\mathrm {CV}=0.293$ to $\mathrm {CV}=0.06$. In the $\mathcal {C}_3$ motif network, a similar behavior was observed, but the autapse-based enhancement strategy of SISR is found to be relatively better in the motif $\mathcal {C}_2$ than $\mathcal {C}_3$ in the sense that $\mathcal {C}_2$ can achieve a lower $\mathrm {CV}$ and larger range of autaptic parameter values in which these low $\mathrm {CV}$ values can be attained.
- For the enhancement strategy of SISR in a motif based on the multiplexing of this motif with another motif, it was found that only electrical multiplexing of two $\mathcal {C}_2$ motifs can enhance the degree of SISR in one of the motifs. In the $\mathcal {C}_2-\mathcal {C}_3$ multiplex configuration, only inhibitory chemical connections between the motifs can enhance the degree of SISR in $\mathcal {C}_3$.

Although inhibitory connections between groups of neurons are generally thought to support competitive learning (Rumelhart and Zipser 1985; Grossberg 1987; Rabinovich et al. 2001; Savin et al. 2010; Krotov and Hopfield 2019), our finding that inhibitory chemical point-to-point connections between different motifs can enhance the degree of SISR in one of the motifs additionally suggests a putative mechanism for dynamically adjusting neural dynamics to maintain optimal information processing.

In this paper, we treated the input noise process as solely Gaussian. Looking forward, we must be cognizant that Gaussian white noise is only one possible type of a noise which can induce resonance. Stochastic processes with a non-Gaussian distribution are well-known to more accurately model the dynamics of real biological neurons (Wu et al. 2017). In Segev et al. (2002), a plot of interspike interval and interevent interval distributions indicates that neurons and neural network activities are characterized by a non-Gaussian heavy-tail interval distribution, thereby providing a solid reason as to why it makes sense to consider non-Gaussian noise such as Lévy noise in the study of neural systems. Therefore, the mechanism via which noise with a non-Gaussian distribution and a temporal correlation (i.e., colored noise) can induce SISR is worth investigating in future research. In particular, the additional timescale brought into the system by the temporal correlation may come along with new interesting dynamics.

## Data Availability

The simulation data that support the findings of this study are available within the article.
